# Bioinformatic and Phenotypic Analysis of *AtPCP-Ba* Crucial for Silique Development in *Arabidopsis*

**DOI:** 10.3390/plants13182614

**Published:** 2024-09-19

**Authors:** Guangxia Chen, Xiaobin Wu, Ziguo Zhu, Tinggang Li, Guiying Tang, Li Liu, Yusen Wu, Yujiao Ma, Yan Han, Kai Liu, Zhen Han, Xiujie Li, Guowei Yang, Bo Li

**Affiliations:** 1 Shandong Academy of Grape, Jinan 250100, China; cguangxia2004@126.com (G.C.); shanhong98@163.com (Z.Z.); weifengluolu@126.com (T.L.); 15153871569@163.com (L.L.); senwy886@163.com (Y.W.); yujiaoma91@163.com (Y.M.); hanyan_saas@163.com (Y.H.); liukai2429@163.com (K.L.) hanzhen86@163.com (Z.H.); lixiujie-2007@163.com (X.L.); guoweiyang66@163.com (G.Y.); 2 State Key Laboratory of Nutrient Use and Management, Key Laboratory of Agro-Environment of Huang-Huai-Hai Plain, Ministry of Agriculture and Rural Affairs, Institute of Agricultural Resources and Environment, Shandong Academy of Agricultural Sciences, Jinan 250100, China; xbwuferguson@163.com; 3 Institute of Crop Germplasm Resources, Shandong Academy of Agricultural Sciences, Jinan 250100, China; guiyingtang88306@163.com; 4 State Key Laboratory of Nutrient Use and Management, Shandong Academy of Agricultural Sciences, Jinan 250100, China

**Keywords:** silique width, CRPs, signal pathway, *TPD1*, bioinformatic analysis

## Abstract

Silique development exerts significant impacts on crop yield. CRPs (Cysteine-rich peptides) can mediate cell–cell communication during plant reproduction and development. However, the functional characterization and regulatory mechanisms of CRPs in silique development remain unclear. In this study, we identified many CRP genes downstream of the CRP gene *TPD1 (TAPETUM DETERMINANT1)* during silique development using a microarray assay. The novel *Arabidopsis thaliana* pollen-borne CRPs, the PCP-Bs (for pollen coat protein B-class) gene *AtPCP-Ba*, along with *TPD1*, are essential for silique development. The *AtPCP-Ba* was significantly down-regulated in *tpd1* flower buds but up-regulated in *OE-TPD1* flower buds and siliques. The silencing of *AtPCP-Ba* compromised the wider silique of *OE-TPD1* plants and inhibited the morphology of *OE-TPD1* siliques to the size observed in the wild type. A total of 258 CRPs were identified with the bioinformatic analysis in *Arabidopsis*, *Brassica napus*, *Glycine max*, *Oryza sativa*, *Sorghum bicolor*, and *Zea mays*. Based on the evolutionary tree classification, all CRP members can be categorized into five subgroups. Notably, 107 CRP genes were predicted to exhibit abundant expression in flowers and fruits. Most cysteine-rich peptides exhibited high expression levels in *Arabidopsis* and *Brassica napus*. These findings suggested the involvement of the CRP *AtPCP-Ba* in the *TPD1* signaling pathway, thereby regulating silique development in *Arabidopsis*.

## 1. Introduction

Silique morphology plays a crucial role in determining crop yields. In *Arabidopsis thaliana*, the silique consists of multiple fertilized ovules and three main regions, including the valve, replum, and valve margin [1,2]. The silique development originates from a gynoecium comprising two fused carpels connected by a central tissue named the septum [3]. This developmental process encompasses the initiation, growth, maturation, and ripening of the silique set. Silique growth involves both cell division and expansion [4], which commences shortly after fertilization and determines the final silique size [5].

The genetic networks contributing to silique development in *Arabidopsis* have also been elucidated. The YABBY transcription factor gene *CRABS CLAW* [6], the basic helix–loop–helix (bHLH) transcription factor gene *SPATULA* [7], and the auxin response factor *ETTIN* [8] have been identified to influence the carpel morphogenesis. Furthermore, the MADS-box family members *SHATTERPROOF 1* and *2 (SHP1/SHP2)* and *FRUITFUL (FUL)* can play crucial roles in regulating fruit patterning, lignin deposition, cell expansion, and cell separation processes within the silique [9,10,11]. Mutants of *REPLUMLESS (RPL)* exhibit a reduced replum width, indicating a pivotal role for RPL in replum development [12]. The downstream regulation of *SHP* involves *INDEHISCENT (IND)* and *ALCATRAZ (ALC)*. *FUL* acts as a repressor of *SHP* and *IND* expression at the valves, while *RPL* functions similarly in the replum region. *FUL* and *RPL* can restrict the expression of *SHP*, *ALC*, and *IND* to narrow strips of cells at the valve margins [13]. The *FUL–SHP* pathway is crucial in silique morphology and evolutionarily conserved across plants [14]. Several signaling molecules, including GA, ethylene, auxin, and cytokinin, along with their intricate interactions, have been recognized as regulators of silique formation and growth [15]. The signals derived from male and female gametophytes and other vegetative tissues are known to promote silique development [16]. Nevertheless, the mechanisms by which the gametophyte-related genes and signals influence silique development remain unknown.

Plant small signaling peptides are a class of small peptides with a protein length of less than 180 amino acids. As novel and important signaling molecules, they play an important role in plant development and responses to stresses [17]. Cysteine-rich peptides (CRPs) are one crucial class of plant small signaling peptides in various aspects of plant defense, growth, development, and reproduction [18]. They typically exhibit three common structural features, namely a small protein length, typically under 160 amino acids, a conserved secretory signal peptide at the N-terminus, and a cysteine-rich domain at the C-terminus, usually containing four to sixteen cysteine residues [19]. Various cysteine-rich peptides, including the RAPID ALKALINISATION FACTOR (RALF), EPIDERMAL PATTERNING FACTOR (EPF), and EPF-LIKE (EPFL), have been reported to function in reproduction and development. The diverse RALF peptides have different cellular functions depending on their interactions with various receptors [20]. Specifically, the RALF coordinates with its receptor BRI1-associated receptor kinase1 (BAK1) to regulate root growth and hair initiation [21]. The RALF–FERONIA signaling pathway involves numerous cellular processes and stress responses, including Ca accumulation, H-ATPase activity, stomatal movement, flowering time, salinity tolerance, and immune response [22,23]. Members of the EPF/EPFL family, such as EPF2 and EPFL9, competitively bind to their co-receptors, ERECTA (ER) and TOO MANY MOUTHS (TMM), modulating stomatal patterning [24]. EPFL2 and EPFL9 are involved in ovule patterns, thereby regulating seed number with gynoecium and fruit growth by binding to ERECTA (ER)-family Leucine-Rich Repeat Receptor-Like Kinases (LRR-RLKs) [25]. However, research on the involvement of CRPs in *Arabidopsis* silique development remains limited.

TPD1, a small cysteine-rich peptide, and the LRR-RLK EXCESS MICROSPOROCYTES1 (EMS1, also known as EXTRA SPOROGENOUS CELLS, EXS) are essential for cell specification and proliferation during anther development in *Arabidopsis*. The *AtTPD1* mutation results in the absence of tapetal cells and pollen grains, leading to complete male sterility, a phenotype consistent with *ems1* and *tpd1 ems1* double mutants [26,27]. TPD1, serving as a peptide ligand, directly interacts with EMS1, triggering the phosphorylation of the EMS1 kinase domain to determine the somatic and reproductive cell fate in *Arabidopsis* anthers [28,29]. Moreover, ectopic overexpression of *TPD1* could induce carpel cell division and alter the silique morphology in *Arabidopsis* [30,31]. The maize MAC1 and rice TDL1A, which are orthologs of TPD1, are also crucial signal peptides in their respective species [32]. Therefore, exploring the TPD1–EMS1 signaling pathway in both monocots and dicots is imperative. However, the downstream signaling components of the TPD1–EMS1 pathway that regulate the cell fate and division during *Arabidopsis* development remain unclear.

Exploring and identifying the cysteine-rich peptide-encoding genes in siliques may provide a foundation for utilizing genetic engineering techniques to enhance crop growth and yield. Four *A. thaliana* PCP-B (pollen coat protein B-class) encoding proteins, namely AtPCP-Ba, AtPCP-Bb, AtPCP-Br, and AtPCP-Bꝺ, as important regulatory small peptides, establish a molecular dialog between the stigma and pollen grains during the earliest stages of the pollen–stigma interaction [33]. Phenotypic analysis revealed defective pollen hydration and delayed pollen tube growth in *pcp-b* mutants compared to wild-type ones. Triple mutant *pcp-ba/b/r* pollen showed considerably reduced hydration on the stigma surface, a weaker anchorage to the stigma surface, and slow pollen tube growth [33]. In this study, we isolated and identified the cysteine-rich peptide gene *AtPCP-Ba* from a microarray analysis of the *tpd1* mutant and *OE-TPD1* plants and investigated the relationship between *AtPCP-Ba* and *TPD1* in silique development. A knock-down of *AtPCP-Ba* compromised the wider silique of *OE-TPD1* plants, resulting in the restoration into wild-type siliques. Additionally, we conducted an evolutionary tree classification of 258 CRPs across *Arabidopsis*, *Brassica napus*, *Glycine max*, *Oryza sativa*, *Sorghum bicolor*, and *Zea mays*. Among them, 107 CRP genes were predicted to be abundant in flowers and fruits, with many showing high expression levels in *Arabidopsis* and *Brassica napus*. These findings suggested the potential involvement of the CRP *AtPCP-Ba* in the *TPD1* signaling pathway, thereby regulating silique development in *Arabidopsis*.

## 2. Results

### 2.1. TPD1 Promotes the Expression of AtPCP-Ba Gene during Silique Development

To investigate the *TPD1–EMS1* signaling pathway in silique development, the *OE-TPD1* plants, *tpd1,* and *ems1* homozygous mutants were used to perform microarray analysis. The wider silique phenotype in *OE-TPD1* plants was observed from the 12th stage of flower development (Figure S1). To screen the essential genes downstream of *TPD1* that regulate silique width, the flower buds were selected before the 12th stage of flower development for the microarray assays. Three technical RNA replicates were pooled for each genotype per time point for labeling and hybridization, sourced from three independent biological replicates of the flower buds.

**Figure 1:**
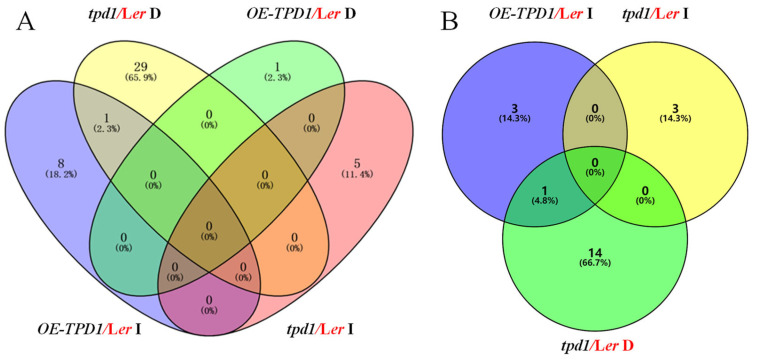
Plant small signaling peptides and cysteine-rich peptides were altered in microarray data. (**A**) Analysis of plant small signaling peptide genes that were altered in the microarray data. (Purple oval: the number and percentage of plant small signaling peptides that were increased in *OE-TPD1* compared with L*er;* green oval: the number and percentage of plant small signaling peptides that were decreased in *OE-TPD1* compared with L*er*; pink oval: the number and percentage of plant small signaling peptides that were increased in *tpd1* compared with L*er*; yellow oval: the number and percentage of plant small signaling peptides that were decreased in *tpd1* compared with L*er*). (**B**) Analysis of cysteine-rich peptide genes, which were altered in the microarray data. Blue circle: the number and percentage of cysteine-rich peptide genes that were increased in *OE-TPD1* compared with L*er*; yellow circle: the number and percentage of cysteine-rich peptide genes that were increased in *tpd1* compared with *Ler*; Green circle: the number and percentage of cysteine-rich peptide genes that were decreased in *tpd1* compared with L*er*. I: increased gene number; D: decreased gene number; the number and percentages in the Venny diagram indicate target gene numbers and percentages in the microarray data.

**Figure 2:**
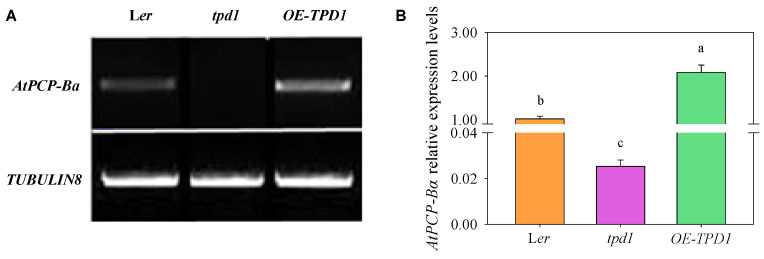
Transcriptional expression analysis of *AtPCP-Ba* in L*er*, *tpd1*, and *OE-TPD1*. (**A**) RT-PCR analysis of *AtPCP-Ba* expression in L*er*, *tpd1*, and *OE-TPD1*; (**B**) real-time PCR analysis of *AtPCP-Ba* expression in L*er*, *tpd1*, and *OE-TPD1*. The RNA was extracted from flower buds including the carpel before the 12th flower development in L*er*, *tpd1*, and *OE-TPD1* plants to analyze *AtPCP-Ba* expression levels. Different letters denote significant differences (LSD, *p* ≤ 0.05) between *AtPCP-Ba* expression in three different plants’ flower buds.

A total of 44 small signaling peptide genes were found to be differentially expressed between wild-type plants and each genotype plant with a false discovery rate (FDR) < 0.05, including nine that were up-regulated in *OE-TPD1,* one that was down-regulated in *OE-TPD1,* five that were up-regulated in *tpd1* and 30 that were down-regulated in *tpd1* by at least twofold (Figure 1A, Excel S1). Notably, among these 44 small signaling peptide genes were 21 cysteine-rich peptides (CRPs), including four (19.1%) CRPs up-regulated in *OE-TPD1*, three (14.3%) CRPs up-regulated in *tpd1*, and 15 (71.5%) CRPs down-regulated in *tpd1* (Figure 1B). Among these candidates, the *AtPCP-Ba* gene was identified using the microarray analysis of *TPD1* overexpressors and *tpd1* mutants from flower buds. *AtPCP-Ba* exhibited significant up-regulation in *OE-TPD1* flower buds and more than double the down-regulation in *tpd1* and *ems1* mutant flower buds (Table S1). The RT-PCR and real-time PCR confirmed the transcriptional changes in *AtPCP-Ba* and *TPD1* during silique development, with *AtPCP-Ba* notably down-regulated in *tpd1* mutants and up-regulated in the *OE-TPD1* plant flower buds (Figure 2). These findings indicated the role of *TPD1* in promoting *AtPCP-Ba* expression at the transcriptional level.

### 2.2. AtPCP-Ba Is Highly Expressed in Developing Flower Buds

**Figure 3:**
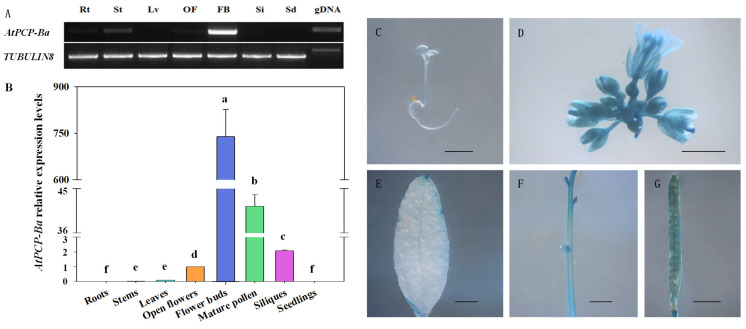
The spatial and temporal expression of *AtPCP-Ba* in *Arabidopsis*. (**A**) RT-PCR assay for the expression of *AtPCP-Ba* in different tissues; (**B**) real-time PCR assay for the expression of *AtPCP-Ba* in different tissues; (**C**–**G**) promoter activity assay in (**C**) seedlings, (**D**) inflorescences, (**E**) leaves, (**F**) stems, and (**G**) siliques of *pAtPCP-Ba::GUS*-transgenic wild plants. Rt, roots (the root of 10 days seedlings); St, stems (the stem of the plants at anthesis); Lv, leaves (the leaves of the plants at anthesis); OF, open flowers (the open flowers of the plants at anthesis); FB, flower buds (the flower buds of the plants at anthesis); Si, siliques (mature siliques); Sd, seedlings (10 day seedlings). Bars = 1 mm (**C**–**G**). The data are represented as mean ± standard error (SE) of three replicates. Statistical significance was determined by one-way analysis of variance; significant differences among means (LSD, *p* ≤ 0.05) are indicated by different lowercase letters.

The RT-PCR and real-time PCR analyses were conducted using the RNAs extracted from various tissues to examine the spatial and temporal expression of *AtPCP-Ba* and validate its relationship with *TPD1*. The findings revealed elevated levels of the *AtPCP-Ba* transcripts in developing flower buds, contrasting with lower levels detected in mature pollen, green siliques, leaves, and stems, whereas expression was scarce in roots and seedlings (Figure 3A,B).

The *AtPCP-Ba* promoter activity assay with a 1 kb sequence in the upstream region was conducted to delineate the spatial and temporal expression profiles of *AtPCP-Ba* (Figure 3C–G). A robust GUS signal was predominantly observed in the flower buds, notably within the anther (Figure 3D). In addition, a weaker GUS signal was detected in siliques, leaves, and stems (Figure 3E–G). Furthermore, the *AtPCP-Ba* expression was predominantly confined to tapetal cells from anther developmental stages 5 to 11, as evidenced by the sectioning of GUS-stained anthers (Figure S2), distinguishing it from TPD1, which is primarily expressed in microsporocytes [27].

### 2.3. Silencing of AtPCP-Ba Compromises the Wider Silique of OE-TPD1 Plants

**Figure 4:**
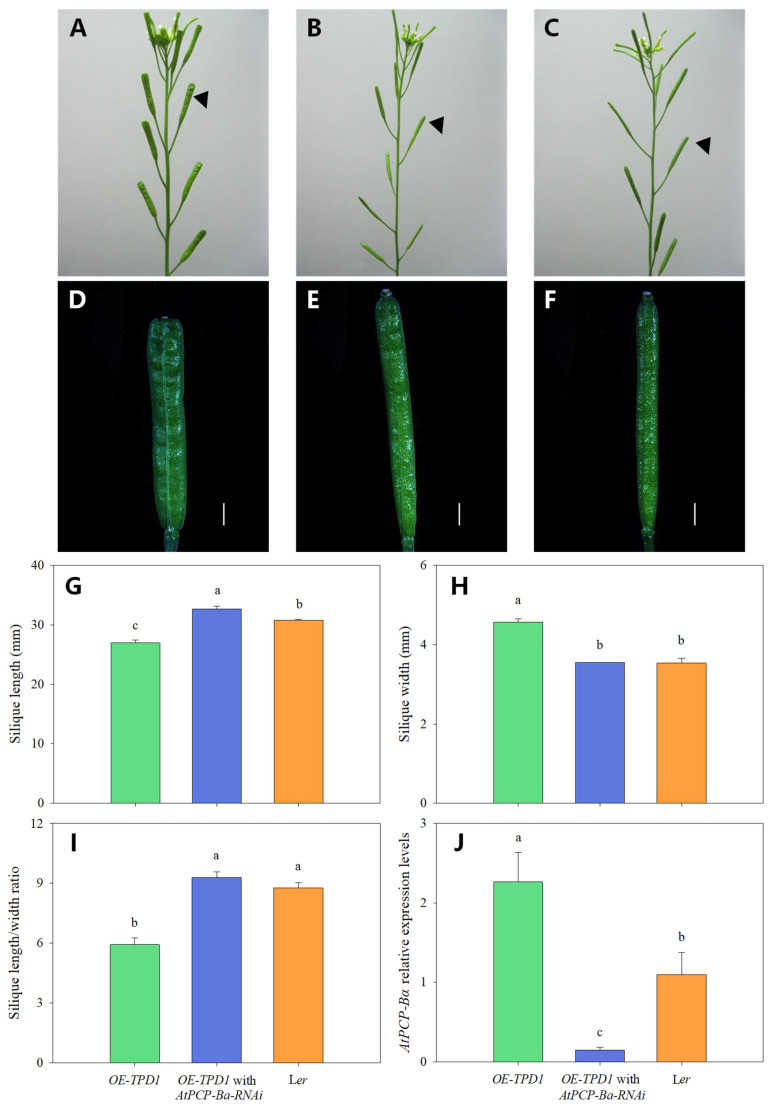
Phenotypic observation of the transgenic *OE-TPD1* plants with the *AtPCP-Ba-RNAi* construct and assay for gene expression. (**A**–**C**) Phenotype of *OE-TPD1* ((**A**) genetic background L*er*), the transgenic *OE-TPD1* with the *AtPCP-Ba-RNAi* construct ((**B**) genetic background *OE-TPD1*), and wild type ((**C**) genetic background L*er*); (**D**–**F**) siliques of *OE-TPD1* (**D**), the transgenic *OE-TPD1* with the *AtPCP-Ba-RNAi* construct (**E**), and wild type (**F**); (**G**–**I**) size observation of siliques of *OE-TPD1*, the transgenic *OE-TPD1* with the *AtPCP-Ba-RNAi* construct, and wild type; (**G**) length measurement of the siliques of *OE-TPD1*, the transgenic *OE-TPD1* with the *AtPCP-Ba-RNAi* construct, and wild type; (**H**) width measurement of the siliques of *OE-TPD1*, the transgenic *OE-TPD1* with the *AtPCP-Ba-RNAi* construct, and wild type; (**I**) ratio analysis of length and width of the siliques of *OE-TPD1*, the transgenic *OE-TPD1* with the *AtPCP-Ba-RNAi* construct, and wild type; (**J**) relative expression level of *AtPCP-Ba* in *OE-TPD1*, the transgenic *OE-TPD1* with the *AtPCP-Ba-RNAi* construct, and wild type; bars = 1 mm (**D**–**F**). Different lowercase letters denote significant differences (LSD, *p* ≤ 0.05) between different silique sizes and gene expression in three different plant mature siliques. The black triangles indicate different siliques from different plants.

**Figure 5:**
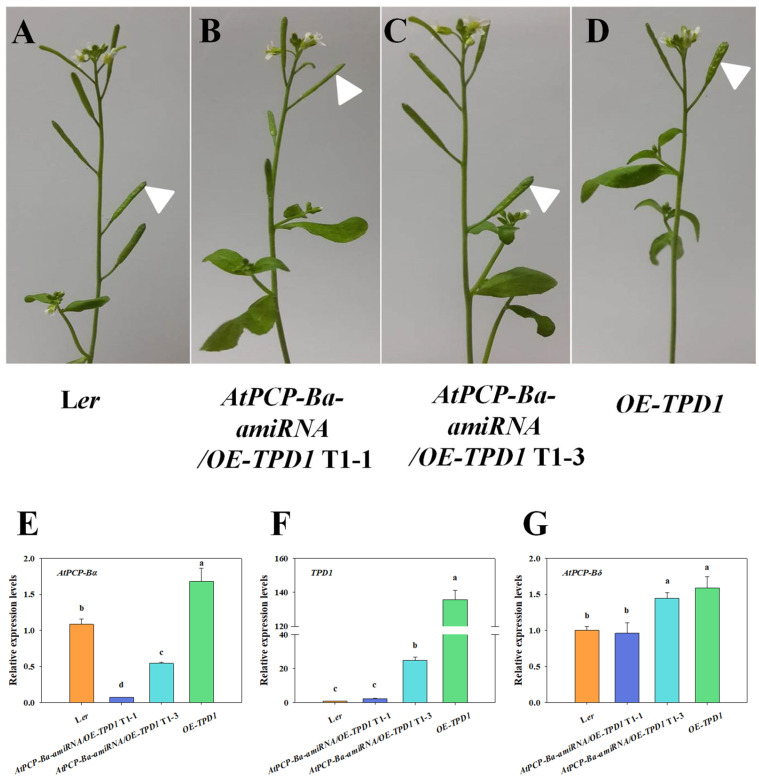
Phenotypic observation of *AtPCP-Ba-amiRNA/OE-TPD1* transgenic plants and assay for gene expression. (**A**–**D**) Phenotypic observation of *AtPCP-Ba-amiRNA/OE-TPD1* transgenic plants; (**E**–**G**) relative expression levels of *AtPCP-Ba*, *TPD1,* and *AtPCP-Bꝺ* in the *AtPCP-Ba-amiRNA/OE-TPD1* transgenic plants. Different lowercase letters denote significant differences (LSD, *p* ≤ 0.05) between different gene expressions in different plant mature siliques. The white triangles indicate different siliques from different plants.

To investigate the role of the *AtPCP-Ba* gene in silique development, two construct vectors, *p35S::AtPCP-Ba* and *AtPCP-Ba*-RNAi, were used to analyze its relationship with *TPD1* during silique development. Both constructs were then transformed into wild-type L*er* plants. However, no notable phenotypic changes were observed in the transgenic plants. The result showed that some obvious silique discrepancy existed in *AtPCP-Ba*-RNAi/*OE-TPD1*-transformed plants when the *AtPCP-Ba*-RNAi was transformed into *OE-TPD1* plants. Some transformed plants showed that the phenotype of *OE-TPD1* siliques partially disappeared, and some transformed plants showed that their silique morphologies are even similar to that of wild-type plants (Figure 4A–F). The siliques of *AtPCP-Ba*-RNAi/*OE-TPD1*-transformed plants were analyzed in length, width, and ratio (Figure 4G–I). The similar reduction in the wider silique phenotype was also observed in the *AtPCP-Ba*-amiRNA/*OE-TPD1* transgenic plants (Figure 5A–D). To make sure the silique morphologies changed, we also adopted an artificial microRNA technique and transformed the new construct *AtPCP-Ba*-amiRNA into *OE-TPD1* plants. The same silique phenotypic changes were observed in *AtPCP-Ba*-amiRNA/*OE-TPD1*-transformed plants. Two lines with different silique morphologies were selected for further analysis (Figure 5B,C). The RNA from the mature siliques was isolated and used for the real-time PCR analysis. The real-time PCR results revealed lower *AtPCP-Ba* gene expression in plants with completely disappeared morphology than in those with wider siliques (Figure 5E). Consistent changes in expression were observed between *AtPCP-Ba* and *TPD1* in both lines (Figure 5F). However, the *AtPCP-Ba* homolog gene *AtPCP-Bꝺ* exhibited no similar changes in expression between the two lines (Figure 5G). These results indicated that *AtPCP-Ba-amiRNA* selectively targeted its intended gene without affecting its homologous counterpart, *AtPCP-Bꝺ*.

### 2.4. Molecular Characterization of AtPCP-Ba in Plants

**Figure 6:**
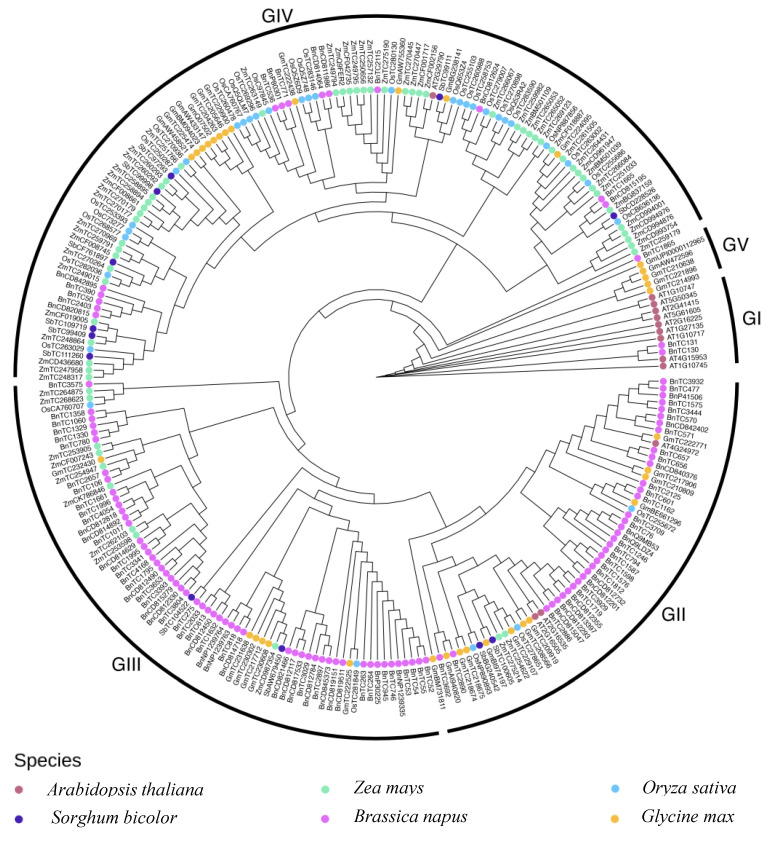
Phylogenetic relationship analysis of cysteine-rich peptides (CRPs) in *Arabidopsis thaliana*, *Zea mays*, *Oryza sativa*, *Sorghum bicolor*, *Brassica napus*, and *Glycine max*. A total of 13 AtCRP genes, 60 ZmCRP genes, 33 OsCRP genes, 12 SbCRP genes, 35 GmCRP genes, and 105 BnCRP genes were clustered into GI, GII, GIII, GIV, and GV groups. The GI group contains AtPCP-Ba (AT5G61605), 8 AtCRPs, and 2 BnCRPs. GII, GIII, GIV, and GV groups encompass 57, 72, 112, and 6 CRP genes, respectively. Full-length amino acid sequences were aligned using MUSCLE, and a tree was constructed using the neighbor-joining (NJ) method in MEGA7.0 software. Each species is depicted in a distinct color.

The AtPCP-Ba protein possessed a typical N-terminal signal peptide domain and a C-terminal sequence featuring seven cysteine residues, confirming its classification in the CRP family (Figure S3). The multiple sequence alignment analysis revealed numerous homologies of *AtPCP-Ba* in several crops, including *Zea mays*, *Oryza sativa*, *Sorghum bicolor*, *Glycine max*, *Brassica napus*, and *Arabidopsis* (Excel S2). The CRPs from the above plants were categorized into five subgroups according to evolutionary tree classification, represented by clades GI, GII, GIII, GIV, and GV (Figure 6). The clades GII, GIII, and GIV encompassed a larger number of family members, comprising 57, 72, and 112 CRP genes, respectively, whereas the clades GI and GV included fewer members, with 11 Arabidopsis CRP family genes and six other CRP genes, respectively (Figure 6). Furthermore, we analyzed their conserved motifs, revealing that AtPCP-Ba shared a high similarity and identical motif 2 composition with numerous CRPs in *Zea mays*, *Oryza sativa*, *Sorghum bicolor*, and *Arabidopsis* (Figure S4C). The presence of motifs 1, 2, and 3 in most CRP proteins indicated their involvement in a conserved domain within CRP proteins (Figure S4C). Additionally, we grouped the CRP genes from monocotyledons and dicotyledons into separate clusters, suggesting independent evolution after the divergence of monocotyledons and dicotyledons (Figure S4).

**Figure 7:**
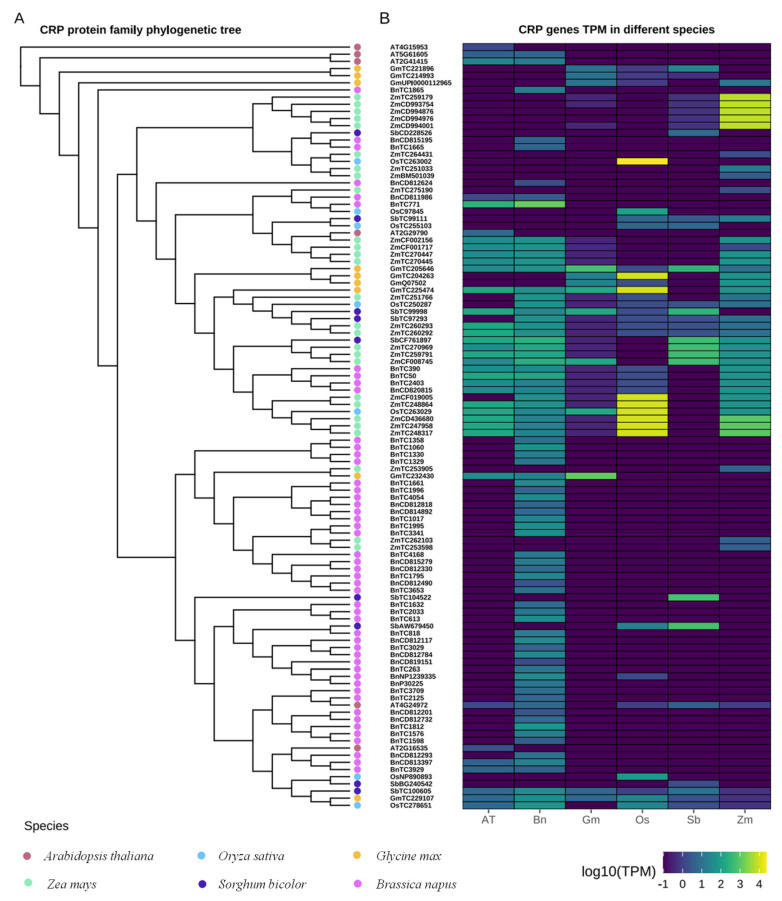
In silico expression analysis of 107 CRPs rich in reproductive organs from six species. A heatmap was generated based on the row scale and log2 fold of fragments per kilobase million (FPKM). (**A**) Phylogenetic tree of 107 CRP genes rich in flowers and fruits from six species; (**B**) heatmap of gene expression patterns. Expression levels of 107 CRP genes in six species were obtained in FPKM of transcriptome analysis.

The analysis was conducted on the 107 CRP genes predicted to be abundant in reproductive organs across the following six species: *Arabidopsis*, *Zea mays*, *Oryza sativa*, *Sorghum bicolor*, *Brassica napus*, and *Glycine max*, utilizing the RNA-seq data obtained from the database (Figure 7). These CRP genes are predicted to be expressed broadly in different species with notable variations among individual genes. Some genes exhibited high expression levels in the reproductive organs of *Oryza sativa* and *Zea mays*. Some genes showed significantly increased expression levels in the reproductive organs of *Arabidopsis* and *Brassica napus*. Notably, *AtPCP-Ba* showed similar expression levels in the reproductive organs of *Arabidopsis* and *Brassica napus* compared to that of other crops (Figure 7B).

### 2.5. Expression Pattern of AtPCP-Ba Homologies Regulated by TPD1 in Different Plants and Tissues

**Figure 8:**
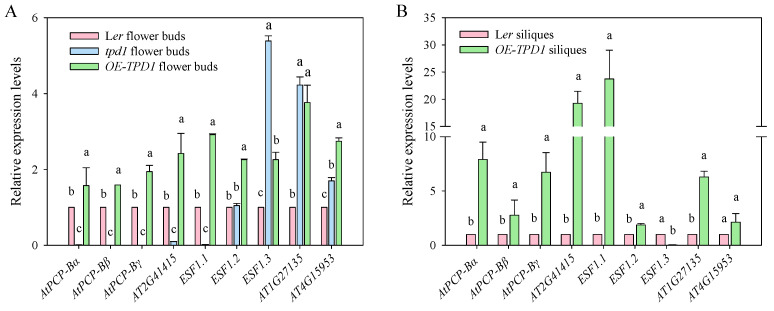
Relative expression levels of *AtPCP-Ba* homologous genes in flower buds before anthesis and mature siliques. (**A**) Real-time PCR assay for expression of *AtPCP-Ba* homologous genes in the wild-type, *tpd1,* and *OE-TPD1* flower buds before anthesis; (**B**) real-time PCR assay for expression of *AtPCP-Ba* homologous genes in the wild-type and *OE-TPD1* mature siliques. Different letters denote significant differences (LSD, *p* ≤ 0.05) between different CRP genes expressions in three different plant flower buds (in (**A**)) or between different CRP genes expressions in three different plants’ mature siliques (in (**B**)).

The expression levels of *AtPCP-Ba*, *AtPCP-Bꝺ*, *AtPCP-Br*, *AT2G41415*, and *ESF1.1* genes were assessed in flower buds of *tpd1* mutants and *OE-TPD1* plants using real-time PCR (Figure 8). Significant decreases were observed in the expression levels of these homologous genes in *tpd1* flower buds compared to those in the wild type. Conversely, the expression levels increased in the flower buds of *OE-TPD1* plants. Notably, no significant change was observed in the expression level of *ESF1.2* in *tpd1* mutant flower buds. However, its expression level was notably higher in *OE-TPD1* plant flower buds than in the wild type. Moreover, increased expression levels were detected for *ESF1.3*, *ATlGl27135*, and *AT4Gl5953* genes in *tpd1* mutant flower buds compared to the wild type. Similarly, their expression levels were significantly elevated in siliques from *OE-TPD1*-overexpressing plants than the wild type (Figure 8A). These alterations were further investigated using siliques from both wild-type and *OE-TPD1* plants, revealing significantly higher expression for all genes, except for the down-regulation observed in the *ESF1.3* gene within silique tissues from *OE-TPD1*-overexpressing plants (Figure 8B). Additionally, *AtPCP-Ba* was also up-regulated in the *OE-TPD1* siliques (Figure 8B). These results indicated the *AtPCP-Ba* and some homologies were indeed regulated by *TPD1* at the transcriptional level.

To further explore the biological functions of the *AtPCP-Ba* homologous genes, we examined their spatial and temporal expression patterns in *Arabidopsis* using real-time PCR (Figure S5). In addition to the prominently expressed *AtPCP-Ba* gene in flower buds, we detected the expression of four genes, including *AtPCP-Bꝺ*, *AtPCP-Br*, *AT2G41415*, and *ESF1.1*, in flower buds, particularly within pollen. *ESF1.1* and *AtPCP-Bꝺ* also exhibited expression levels in siliques (Figure S5). Conversely, the *ESF1.3*, *ESF1.2*, and *AT4G15953* genes displayed higher expression levels in siliques, and *ESF1.3* was also expressed in pollen. Notably, the *ESF1.2* gene expression levels were significantly higher in open flowers and flower buds than in other tissue parts (Figure S5).

## 3. Discussion

Various plant small signaling peptides play vital roles in diverse growth and development processes, including cell proliferation [34,35], mineral element absorption and regulation [36], root development [36,37,38,39,40,41], pollen fertility [42,43,44], stomata aperture regulation [45,46], defense resistance [47,48,49], and environmental adaptation [50]. Cysteine-rich peptides are essential for pivotal reproductive processes, such as self-incompatibility, pollen tube elongation, guidance, and gamete interactions [51]. AtLURE1s and XIUQIUs, cysteine-rich pollen tube attractants, are secreted by synergid cells and diffuse from the micropylar region of the ovule toward the surfaces of the placenta and septum, respectively [52]. Cysteine-rich peptides EPFL2 and EPFL9 regulate ovule patterning and govern seed numbers in gynoecium and fruit growth through shared receptors [25]. Recent research has demonstrated that AtPCP-Ba, AtPCP-Bb, and AtPCP-Br, as important cysteine-rich peptides, establish the dialog between the stigma and pollen grains during the pollen–stigma interaction [33]. The depletion of *PCP-Ba/b/r* significantly slows the process of pollen hydration and germination [33]. Pollen PCP-Bb/r peptides competitively bind to the ANJ–FER receptor complex, displacing RALF23/33 and effectively reducing stigmatic ROS levels to facilitate pollen hydration [53]. Our research has demonstrated that *AtPCP-Ba* was expressed in tapetal cells, which persisted from anther developmental stages 5 to 11 during the tapetal development in *Arabidopsis* (Figure S2). This result suggested that *AtPCP-Ba* may function in pollen development, which is consistent with previous research.

In this study, we discovered a novel function of AtPCP-Ba as a vital CRP in silique morphology development. *AtPCP-Ba*, along with *TPD1*, represented two newly identified cysteine-rich peptide genes implicated in silique development. Our analysis focused on elucidating the relationship between *AtPCP-Ba* and *TPD1*. Using a microarray analysis, we isolated and identified the *AtPCP-Ba* gene by comparing the gene expression in the *tpd1* mutant and *OE-TPD1*. *TPD1* is expressed mostly in developing microsporocytes and is required for the specialization of tapetal cells in the *Arabidopsis* anther [27]. *OE-TPD1* altered the pattern of siliques from the 12th stage of flower development (Figure S1C,D). We speculate that *AtPCP-Ba* with *TPD1* are the signals from male gametophytes to promote silique development. The down-regulation of the *AtPCP-Ba* gene using artificial microRNA and RNAi technology in *OE-TPD1* plants led to decreased expression levels of *TPD1* and weakened or even abolished the phenotype of *OE-TPD1*. Our results confirmed the positive correlation between *AtPCP-Ba* and *TPD1* in the regulation of silique morphology development in *Arabidopsis*. The *AtPCP-Ba* gene expression was up-regulated with *TPD1* up-regulation. Meanwhile, the *AtPCP-Ba* gene expression was down-regulated with the *TPD1* down-regulation. Additionally, *AtPCP-Ba* may exert the feedback regulation of *TPD1*. Because *TPD1* was also decreased when *AtPCP-Ba* gene expression was down-regulated, *TPD1* might undergo feedback regulation by *AtPCP-Ba*, in addition to affecting the expression of the *AtPCP-Ba* gene.

TPD1, a small, secreted cysteine-rich protein ligand, can interact with the LRR (leucine-rich repeat) domain of the EMS1 receptor kinase, influencing tapetum cell fate and carpel development [28]. The ectopic *TPD1* expression induces wider siliques by promoting additional cell division, suggesting that the ectopic expression of TPD1–EMS1 signaling affects cell division [30]. Similar to the TPD1 protein, the AtPCP-Ba protein possesses an N-terminal signal peptide (Figure S3B,C) (https://services.healthtech.dtu.dk/service.php?SignalP-5.0 (accessed on 18 March 2011)). LRR-RLKs and MAPKs play crucial roles in regulating cell division and proliferation in plants [54]. Various ligand–receptor pairs frequently share common downstream signaling components, including mitogen-activated protein kinase (MAPK) signaling networks [55]. The TPD1-activated EMS1 pathway can likely play a significant role in cell division by triggering the MAPK cascade. It is hypothesized that AtPCP-Ba may serve a similar function as a signaling molecule, necessitating the identification of its receptor protein in silique development. Our findings indicated that *TPD1* primarily regulated the expression of *AtPCP-Ba*, which could mitigate the widened silique phenotype of *OE-TPD1*. Notably, the single receptor could perceive multiple peptides, as seen with EPF2 and STOMAGEN/EPF-LIKE 9 (EPFL9), both competitively binding to the ERECTA receptor to regulate stomatal patterning in the leaf epidermis [24,56]. Despite the genetic interaction between *TPD1* and *AtPCP-Ba*, further research is imperative to identify the receptor of AtPCP-Ba, and additional investigation is required to entirely elucidate the relationship between AtPCP-Ba and TPD1, as well as their specific mechanisms of silique development.

The evolutionary relationships among *Arabidopsis*, *Brassica napus*, *Glycine max*, *Oryza sativa*, *Sorghum bicolor*, and *Zea mays* were deduced from the analysis of 258 small cysteine-rich secretory peptides, resulting in the classification of five distinct subgroups. This evolutionary tree was compared using all protein sequences of CRP members, differing from previous alignment data based on predicted mature protein-coding regions [33]. Among these, 107 cysteine-rich peptide genes were predicted to be highly expressed in flowers and fruits. Most cysteine-rich peptides were clustered in *Arabidopsis* and *Brassica napus*. Eleven small CRP proteins demonstrated notable similarities to AtPCP-Ba in *Arabidopsis*. The real-time PCR assay revealed elevated expression levels of *AtPCP-Ba*, *AtPCP-Bꝺ*, *AtPCP-Br*, *AT2G41415*, and *ESF1.1* in flower buds and pollen. *AT1G27135* was highly expressed in pollen, and the other three genes, namely *ESF1.2*, *ESF1.3*, and *AT4G15953*, were expressed in siliques. These CRPs are highly expressed in the reproductive organs of *Arabidopsis* plants. Additionally, the real-time PCR assay demonstrated a significant down-regulation of gene expression levels of *AtPCP-Ba*, *AtPCP-Bꝺ*, *AtPCP-Br*, *AT2G41415*, and *ESF1.1* in *tpd1* mutants, but a significant up-regulation in *OE-TPD1* plants (Figure 8A). These results suggested that *AtPCP-Ba*, along with the other four genes *AtPCP-Bꝺ*, *AtPCP-Br*, *AT2G41415*, and *ESF1.1*, may be associated with the *TPD1* gene in silique development. Some studies have indicated that *ESF1.1*, *ESF1.2*, and *ESF1.3* participate in the membrane-associated receptor-like cytoplasmic kinase SSP (SHORTSUSPENSOR) and the MAPK (mitogen-activated protein kinase) YDA-mediated signal transduction pathway, thereby regulating the early development of *Arabidopsis* embryos [57].

The absence of T-DNA mutants for *AtPCP-Ba* led to the generation of both *amiR-AtPCP-Ba* and *RNAi-AtPCP-Ba* transgenic plants via transformation into wild-type L*er*. However, no identifiable phenotypic differences were observed in L*er* transgenic plants. Nonetheless, a significant reduction in pollen hydration was noted in triple mutants of *AtPCP-Ba*, *AtPCP-Br*, and *AtPCP-Bb* [33]. The high similarity in the amino acid sequences of AtPCP-Ba, AtPCP-Bꝺ, and AtPCP-Br suggested potential functional redundancy in plant development. The artificial microRNA and RNAi technology for the down-regulation of the *AtPCP-Ba* gene in *OE-TPD1* plants led to the weakened or even abolished wide silique phenotype of *OE-TPD1*. The real-time PCR analysis indicated that the expression level of the homologous gene *AtPCP-Bꝺ* was similar to that observed in amiR-*AtPCP-Ba-OE-TPD1* transgenic plants, where morphological changes had completely disappeared compared to wider siliques (Figure 5G). Further investigation revealed an up-regulated expression level of AtCPB-Aꝺ in transgenic plants with wider siliques expressing amiR-*AtPCP-Ba-OE-TPD1* as well as in *OE-TPD1* transgenic plants (Figure 5G). This observation was consistent with the finding that elevated TPD1 expression levels led to higher relative expression levels of both *AtPCP-Ba* and its homologous gene *AtPCP-Bꝺ* in flower buds and siliques from *OE-TPD1* plants (Figure 8), suggesting a potential role for PCP-Ba as a ligand to activate distinct effector targets associated with silique width through TPD1 or to enhance activation via synergistic interactions with putative silique targets. A similar complexity was observed when multiple synergid LUREs collectively functioned through various pollen tube receptors to ensure the proper guidance toward embryo sacs, as seen in species such as *T*. *fournier* and *Arabidopsis* [42,58,59]. The CRPs AtPCP-Ba and TPD1 may function with the same or different carpel receptors to determine the initiation of silique morphology. These findings implied a positive correlation between *AtPCP-Ba* and *TPD1* in cell division and growth during silique development. However, further research is necessary to elucidate the role of *AtPCP-Ba* in silique development. More homologous CRP genes with *AtPCP-Ba* need to be knocked down or out together to observe the silique morphology. More researches about the characterization and functional analysis of CRPs need to be carried out to explicate more signal pathways in silique development. Precise interactions between AtPCP-Ba with other homologous CRPs and their corresponding receptors also need to be identified in silique development. The concentration of CRP required for their physiological functions is extremely low. Unlike traditional plant hormones, CRPs primarily consist of amino acids, thereby posing no environmental risk upon exogenous application. The application of CRPs in agricultural production has the potential to enhance crop yield, thus serving modern green agriculture.

## 4. Materials and Methods

### 4.1. Plant Materials and Growth Conditions

The *Arabidopsis thaliana* plants used in this study originated from a Landsberg *erecta* (L*er*) background. The seeds were surface-sterilized in 8% sodium hypochlorite for 5 min, rinsed three times in sterilized distilled water, and then placed on Murashige and Skoog (MS) salt agar plates before being transferred to a growth chamber at 4 °C for 2 days. Subsequently, the seeds were cultivated in a growth room at 22 °C under a photoperiod of 16 h light and 8 h dark for 7–10 days, then transferred to soil under the same light conditions. Previous studies have documented *OE-TPD1*, *tpd1*, and *ems1* [31]. AmiRNA-*AtPCP-Ba* and RNAi-*AtPCP-Ba* transgenic plants were developed from the L*er* and *OE-TPD1* backgrounds.

### 4.2. Construction of amiRNA-AtPCP-Ba

To construct the amiRNA-*AtPCP-Ba*, RS300 (MIR319a *Arabidopsis thaliana*) served as the backbone. The sequence of the *AtPCP-Ba* gene was analyzed to predict the target of amiRNA in the *Arabidopsis thaliana* genome using Web MicroRNA Designer (http://wmd3.weigelworld.org/cgi-bin/webapp.cgi?page=Designer (accessed on 8 April 2013)). Two separate PCR steps were employed to obtain *AtPCP-Ba* amiRNA fragments. First, pRS300 was used as a template to generate two DNA fragments containing the target gene. Subsequently, another round of PCR was conducted using these two DNA fragments as templates to amplify the full-length DNA fragment amiRNA-*AtPCP-Ba*. The primers used to construct amiRNA-*AtPCP-Ba* are listed in Table S2.

### 4.3. Construction of RNAi-AtPCP-Ba

For obtaining the *AtPCP-Ba* plant RNAi expression vector, the partial coding region of *AtPCP-Ba* was amplified from the pMD18-*AtPCP-Ba* plasmid containing the full-length *AtPCP-Ba* cDNA, using the gene-specific primers RNAi-CDS-F and RNAi-CDS-R (Table S3). The forward primer RNAi-F incorporated NcoI and SwaI restriction enzyme sites at its 5′ end, whereas the reverse primer RNAi-R included XbaI and BamHI restriction enzyme sites at its 3′ end. The digested *AtPCP-Ba* fragments were then inserted into the XbaI/BamHI and NcoI/SwaI enzyme sites of the binary vector pFGC5941 at inverted repeat sequences, resulting in the plant RNAi expression vector pFGC-*AtPCP-Ba*, which was capable of forming a hairpin RNAi construct. The primers used to construct the *AtPCP-Ba*-RNAi are listed in Table S3.

### 4.4. RT-PCR and Real-Time PCR Assays

Total RNAs from various *Arabidopsis* tissues, including roots, shoots, leaves, flowers, flower buds, mature pollen, and seedlings, were extracted using a TRIzol reagent kit (266412, Invitrogen, Carlsbad, CA, USA). The RNA from the siliques was isolated using a total RNA extraction kit (BioTeke, Beijing, China). The SuperScript III First-Strand Synthesis System (18064-014, Invitrogen, CA, USA) was used to synthesize first-strand cDNA according to the manufacturer’s instructions. The cDNA pools from different tissues were used to analyze expression patterns, whereas those from flower buds in mutant and wild-type plants were compared for *AtPCP-Ba* expression levels. Amplifications were performed for 35 cycles for *TUB*, *TPD1*, and *AtPCP-Ba*, with *Tubulin-8* serving as an internal control for RT-PCR. The real-time PCR assays were conducted using a 2×Power SYBR Green PCR Master Mix (4367659, Applied Biosystems, Waltham, MA, USA, www.appliedbiosystems.com) with gene-specific primers on an ABI 7500 real-time instrument (Applied Biosystems, www.appliedbiosystems.com). The PCR program consisted of an initial denaturation step at 95 °C for 10 min, followed by 40 cycles at 95 °C for 15 s and 60 °C for 1 min. After each run, a dissociation curve was generated by gradually heating the samples from 60 °C to 95 °C to confirm amplification specificity. The relative expression levels were determined using the ΔΔCt (threshold cycle) method and normalized to that of *ACTIN2/8* within the same cDNA samples, which were assessed in triplicate for three biological replicates. All primers used in this study are listed in Table S3.

### 4.5. Microarray Analysis

The RNA samples used for microarray assays were extracted from flower buds of wild-type, *OE-TPD1*, *tpd1*, and *ems1-2* homozygous mutants. ATH1 Genome Arrays were employed to compare their transcriptomes. The transcription levels between the wild-type and mutant plants were quantified in triplicate using the ATH genome chips (Affymetrix, http://www.affymetrix.com (accessed on 8 May 2010)). The genes exhibiting expression changes greater than two-fold were categorized as either up-regulated or down-regulated and were selected as candidates for regulation by *TPD1*. The candidates were identified based on fold changes exceeding two or less than 0.5 compared to the wild type.

The total RNA was extracted for microarray experiments, and the RNA quality was assessed by running a 2 μg RNA aliquot on the agarose gel. Sixty micrograms of total RNAs were purified using the Qiagen RNAeasy Mini Kit (Qiagen, Valencia, CA, USA) and used for subsequent experiments. The cDNA synthesis, sample labeling, array hybridization, scanning, and data processing were performed as previously described [60].

### 4.6. GUS Assay

The promoter fragments of *AtPCP-Ba* were amplified using rTaq (DR100A, TaKaRa, Shiga, Japan) and subcloned upstream of the GUS reporter gene in the pCAMBIA1300 Ti-derived binary vector, followed by transformation into wild-type plants. Plant transformation and GUS activity analyses were conducted as previously described [61]. The GUS activity was assessed by staining different transgenic plant tissues in a solution containing 100 mmol/L NaPO_4_ (pH 7.0), 0.5 mmol/L potassium ferricyanide [K_3_Fe(CN)_6_], 0.5 mmol/L potassium ferrocyanide [K_4_Fe(CN)_6_], 0.1% Triton X-100, 10 mmol/L EDTA, and 0.5 mg/mL bromochloroindoyl-β-glucuronide [62]. The staining was performed at 37 °C for 2–3 h, followed by overnight incubation in an acetic acid/ethanol solution (1:3 [*v*/*v*]). The GUS-stained tissues were examined using a Leica DM2500 microscope equipped with a DIC system and MZ10F stereo microscope (Leica, Wetzlar, Germany). The 1.0 kb promoter region upstream of the start codon of *AtPCP-Ba* was fused to the glucuronidase (GUS) reporter gene and introduced into wild-type *Arabidopsis* plants, resulting in the generation of twenty-one independent p*AtPCP-Ba*::GUS transgenic plants.

### 4.7. Light Microscope

The flowers of both wild-type and mutant plants were fixed overnight in an FAA solution comprising 90% ethanol, 5.0% glacial acetic acid, and 5.0% formaldehyde, followed by a 30 min exhaustion process. Subsequently, all the tissues were dehydrated in an ethanol series (70%, 80%, 90%, 95%, and 2 × 100%) for 30 min per concentration, followed by clearing in dimethylbenzene and embedding in paraffin. Dewaxed specimens were sectioned (7 mm) using a microtome (LEICA RM2265, Germany). Anther transverse sections were stained with 0.5% safranin at 37 °C for 40 min and 0.5% Fast Green at room temperature for 1 min. All anther cross-sections were photographed using a microscope (LEICA DM2500, Germany).

### 4.8. Multiple Sequence Alignment and Phylogenetic Analysis

An unrooted phylogenetic tree was constructed using protein sequences from 13 AtCRP proteins in *Arabidopsis*, 60 ZmCRP proteins in *Zea mays*, 33 OsCRP proteins in *Oryza sativa*, 12 SbCRP proteins in *Sorghum bicolor*, 35 GmCRP proteins in *Glycine max*, and 105 BnCRP protein sequences from *Brassica napus* using the neighbor-joining method in MEGA 7.0. The expression patterns of CRP genes in these six species were analyzed using RNA-seq data obtained from relevant databases. The transcription levels of 107 CRP genes across *Arabidopsis*, *Zea mays*, *Oryza sativa*, *Sorghum bicolor*, *Brassica napus*, and *Glycine max* were then analyzed and visualized.

The CRP sequences from various crops were obtained from complete plant genomes using TBLASTN database searches (PHYTOZOME, https://phytozome.jgi.doe.gov (accessed on 22 September 2021); Comparative Genomics, COGE, https://genomevolution.org (accessed on 18 December 2022)), ensuring the completion up to at least the scaffold level. The *Arabidopsis* CRP protein sequences were sourced from *Arabidopsis* Information Resource 4, and the alignment of CRP protein sequences from crops and *Arabidopsis* was performed using the MUSCLE method [63]. Subsequently, an unrooted phylogenetic tree was constructed using the neighbor-joining (NJ) method with a p-distance model and 1000 bootstrap repeats in MEGA 7.0 [64].

## 5. Conclusions

In summary, this study elucidated a novel role of AtPCP-Ba as a pivotal small signal peptide in the development of silique morphology. The intimate association between *AtPCP-Ba* and *TPD1* in silique development, along with their broad similarity to other CRP regulatory proteins, strongly suggested their involvement in interacting with unknown carpel targets to activate silique morphology development. Furthermore, the maintenance and diversity of CRP proteins in *Arabidopsis* and *Brassicaceae* implied their potential contribution to silique development and crop seed yield.

## Data Availability

Transcriptome data under the abiotic stress and biotic treatment are available from the corresponding author (sdtalibo@163.com) upon reasonable request.

## Supplementary Materials

The following supporting information can be downloaded at: https://www.mdpi.com/article/10.3390/plants13182614/s1, Figure S1: The silique phenotypic observation of the *OE-TPD1* and wild type plants; Figure S2: *AtPCP-Ba* was expressed in tapetal cells; Figure S3: AtPCP-Ba protein contains a N-terminal signal peptide; Figure S4: Phylogenetic tree (A), gene structure (B), and conserved motif (C) of flower and silique CRP protein sequences in *Arabidopsis*, *Zea mays*, *Oryza sativa* and *Sorghum bicolor*; Figure S5: Expression patterns of *PCP-B* family genes; Table S1: The microarray assay showed that the expression of *AtPCP-Ba* was significantly affected in *OE-TPD1*, *tpd1,* and *ems1* mutants; Table S2: The primers used in the artificial microRNA assays; Table S3: The primers used in the related vector construct, real-time PCR and RT-PCR assays; Excel S1: All the small signaling peptide genes were found to be differentially expressed between wild-type plants and each genotype plant in the microarray; Excel S2: All the cysteine-rich peptides (CRPs) for phylogenetic relationship analysis in *Arabidopsis*, *Zea mays*, *Oryza sativa*, *Sorghum bicolor*, *Brassica napus*, and *Glycine max*.
