# Supplementary material for: Bioinformatic and Phenotypic Analysis of AtPCP-Ba Crucial for Silique Development in Arabidopsis
Source: Plants (Basel). 2024 Sep 19;13(18):2614. doi: 10.3390/plants13182614 (PMC11435202; doi:10.3390/plants13182614)
Supplement: Supplementary file 1 [file plants-13-02614-s001.zip › Supplementary materials.pptx]

## Slide 1
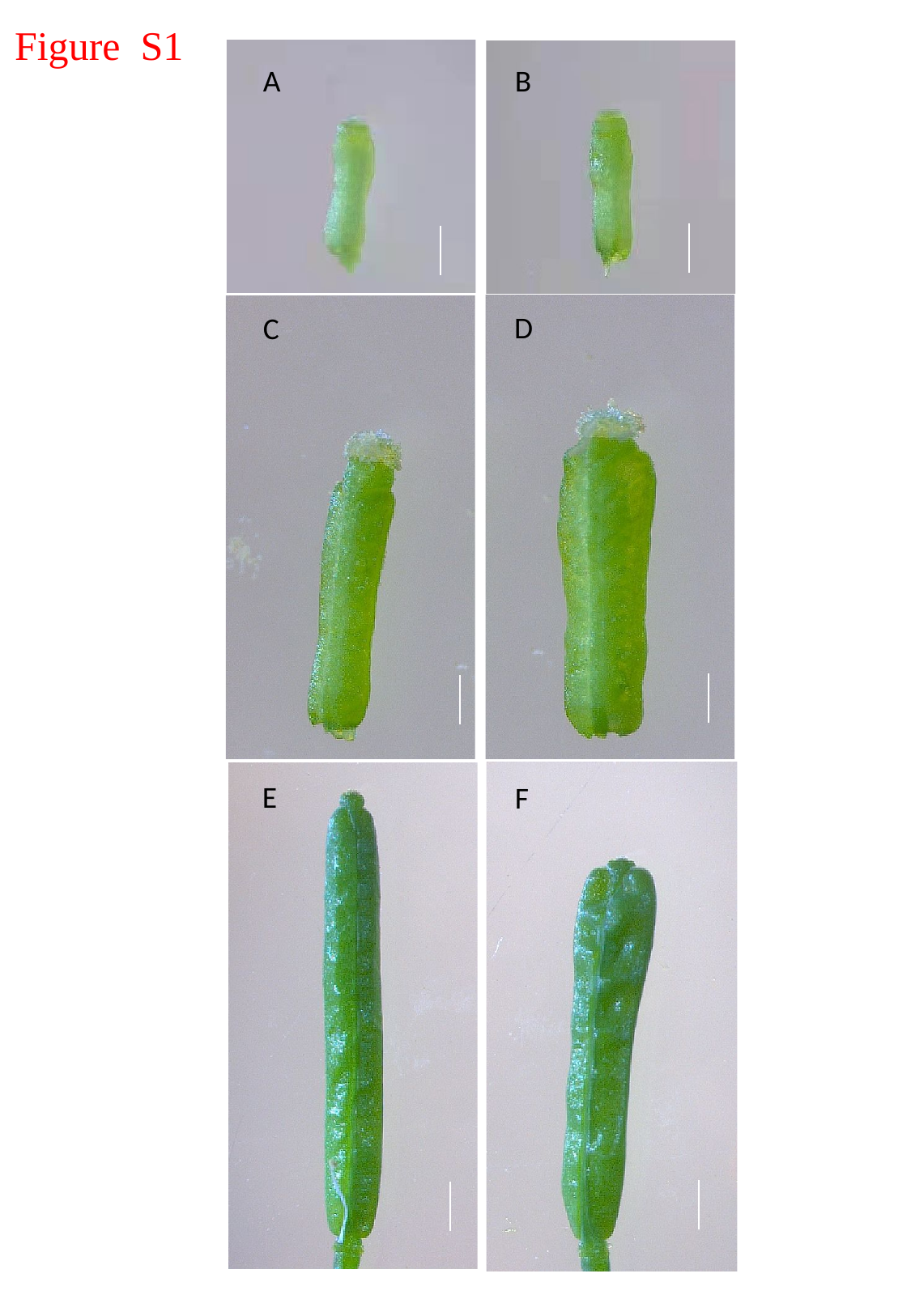

Figure S1
A
B
D
C
E
F

## Slide 2
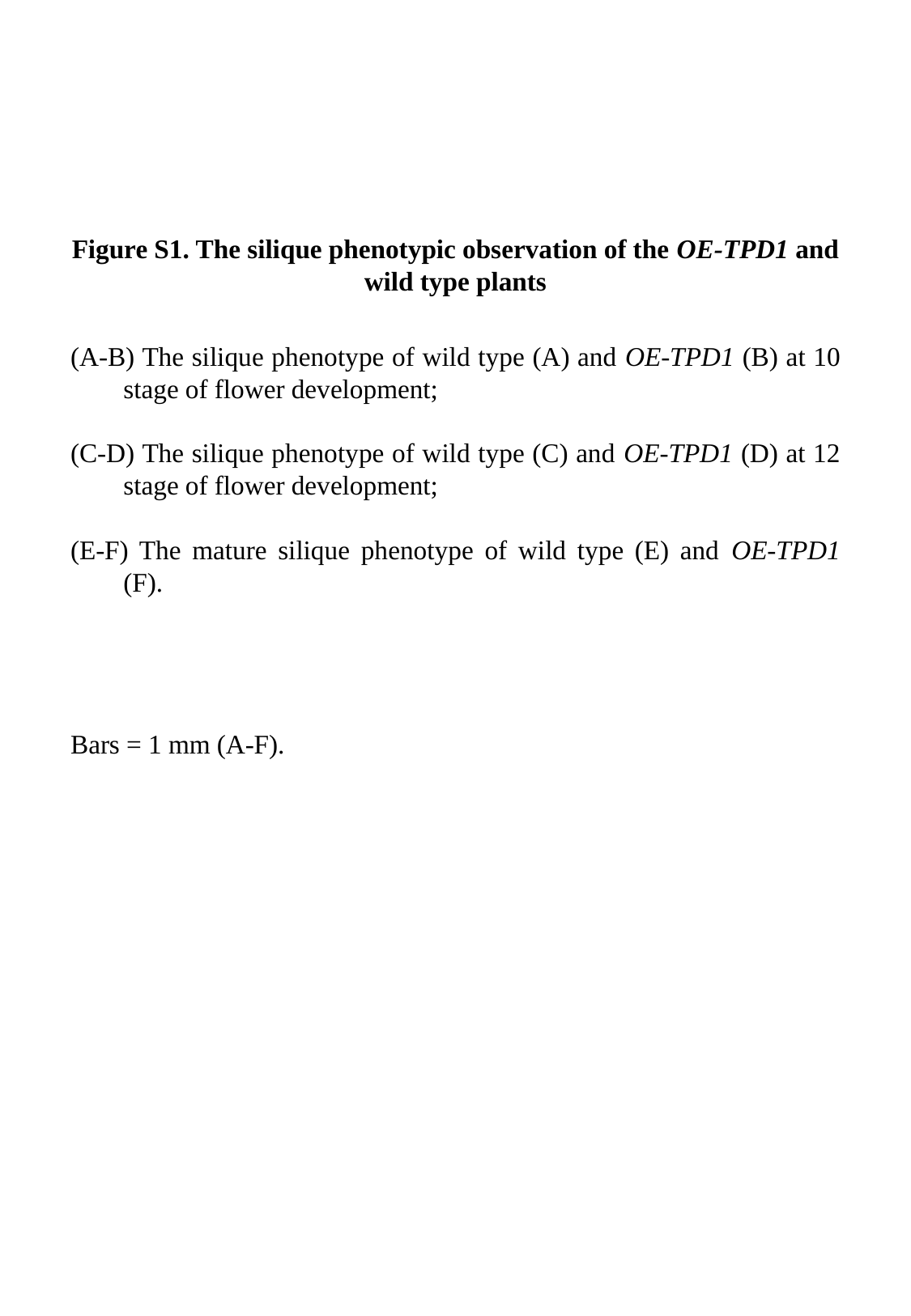

Figure S1. The silique phenotypic observation of the OE-TPD1 and wild type plants
(A-B) The silique phenotype of wild type (A) and OE-TPD1 (B) at 10 stage of flower development;
(C-D) The silique phenotype of wild type (C) and OE-TPD1 (D) at 12 stage of flower development;
(E-F) The mature silique phenotype of wild type (E) and OE-TPD1 (F).
Bars = 1 mm (A-F).

## Slide 3
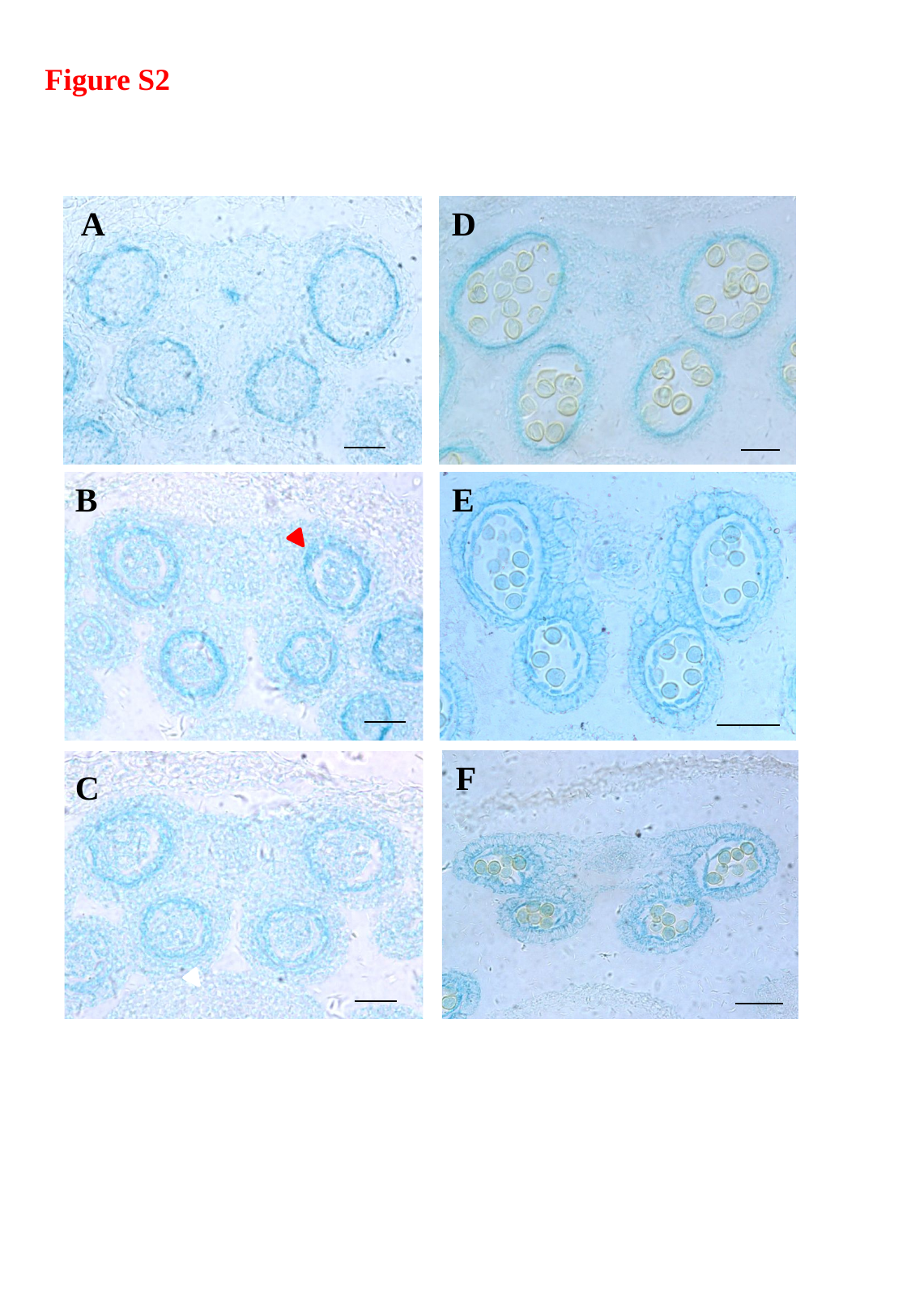

Figure S2
A
D
E
B
C
F

## Slide 4
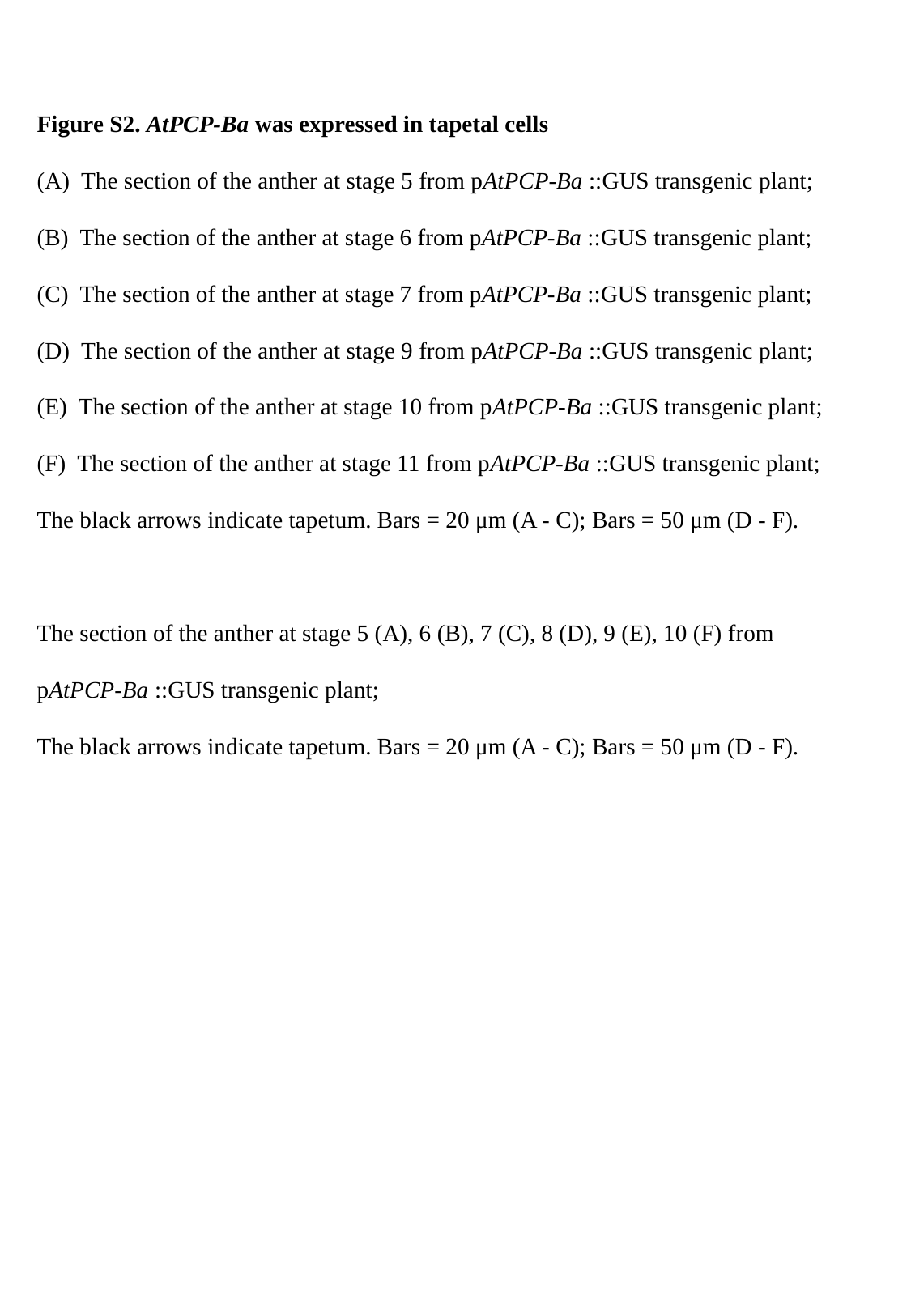

Figure S2. AtPCP-Ba was expressed in tapetal cells
(A) The section of the anther at stage 5 from pAtPCP-Ba ::GUS transgenic plant;
(B) The section of the anther at stage 6 from pAtPCP-Ba ::GUS transgenic plant;
(C) The section of the anther at stage 7 from pAtPCP-Ba ::GUS transgenic plant;
(D) The section of the anther at stage 9 from pAtPCP-Ba ::GUS transgenic plant;
(E) The section of the anther at stage 10 from pAtPCP-Ba ::GUS transgenic plant;
(F) The section of the anther at stage 11 from pAtPCP-Ba ::GUS transgenic plant;
The black arrows indicate tapetum. Bars = 20 μm (A - C); Bars = 50 μm (D - F).
The section of the anther at stage 5 (A), 6 (B), 7 (C), 8 (D), 9 (E), 10 (F) from
pAtPCP-Ba ::GUS transgenic plant;
The black arrows indicate tapetum. Bars = 20 μm (A - C); Bars = 50 μm (D - F).

## Slide 5
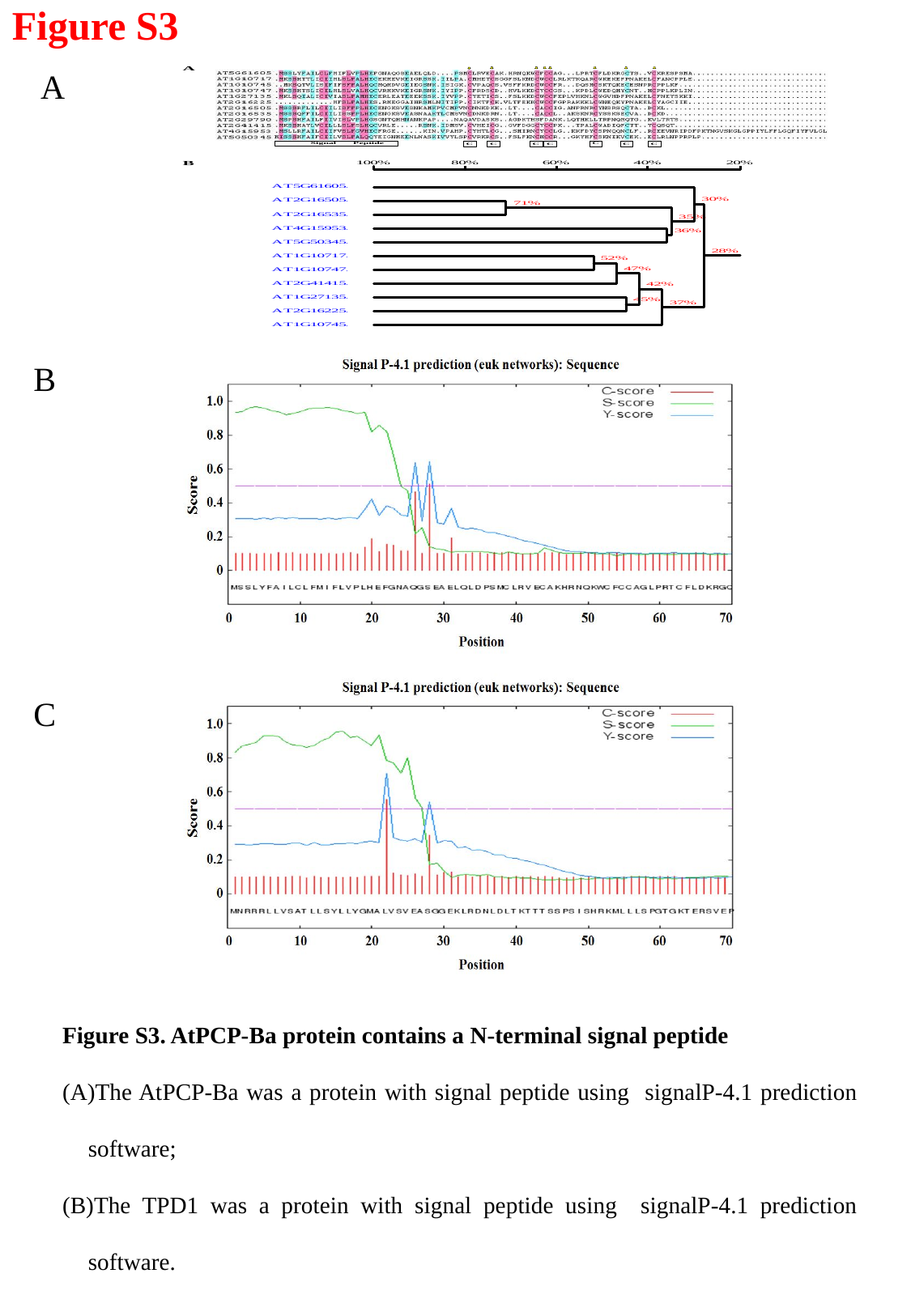

Figure S3
A
B
C
Figure S3. AtPCP-Ba protein contains a N-terminal signal peptide
The AtPCP-Ba was a protein with signal peptide using signalP-4.1 prediction software;
The TPD1 was a protein with signal peptide using signalP-4.1 prediction software.

## Slide 6
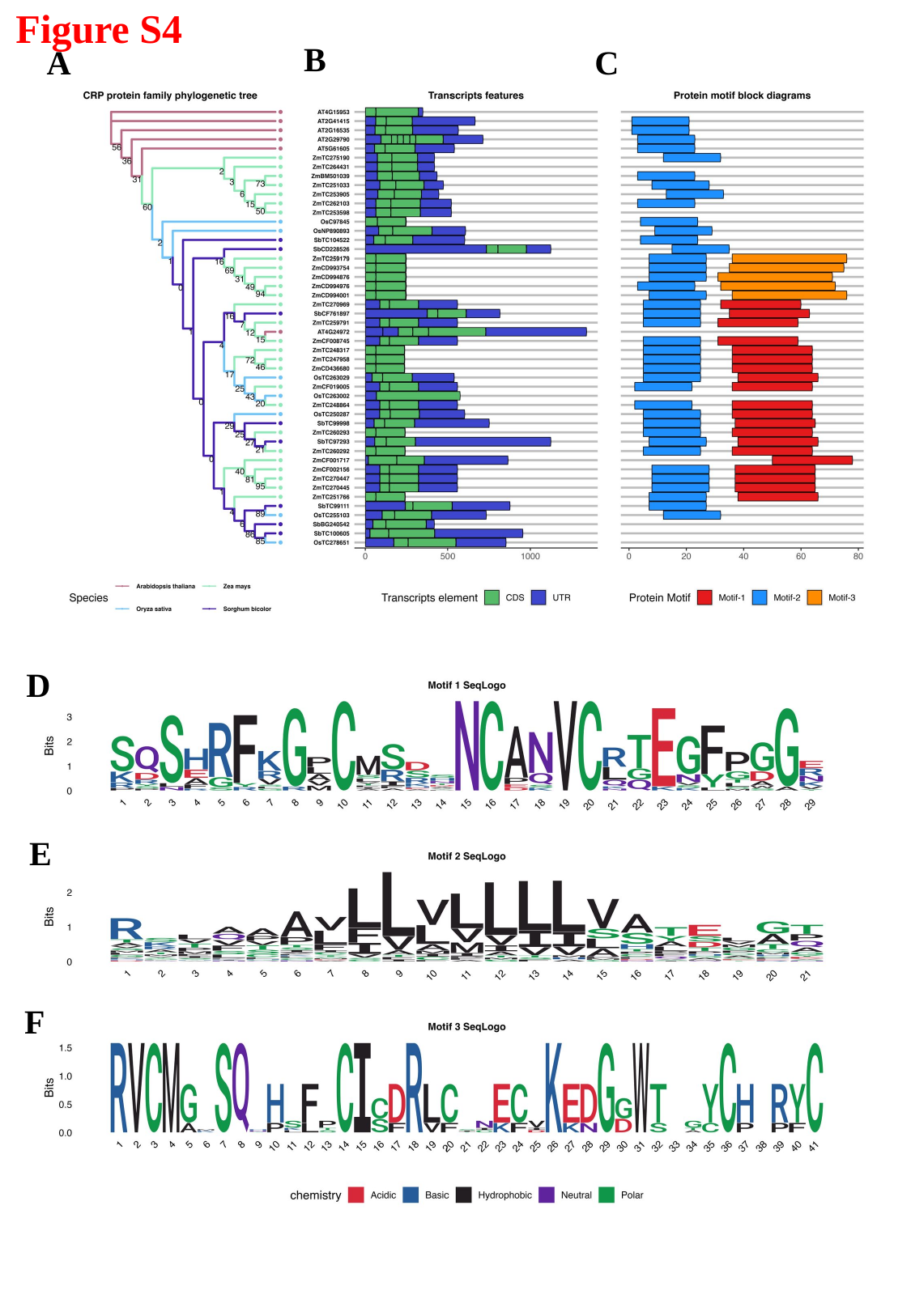

Figure S4
B
A
C
D
E
F

## Slide 7
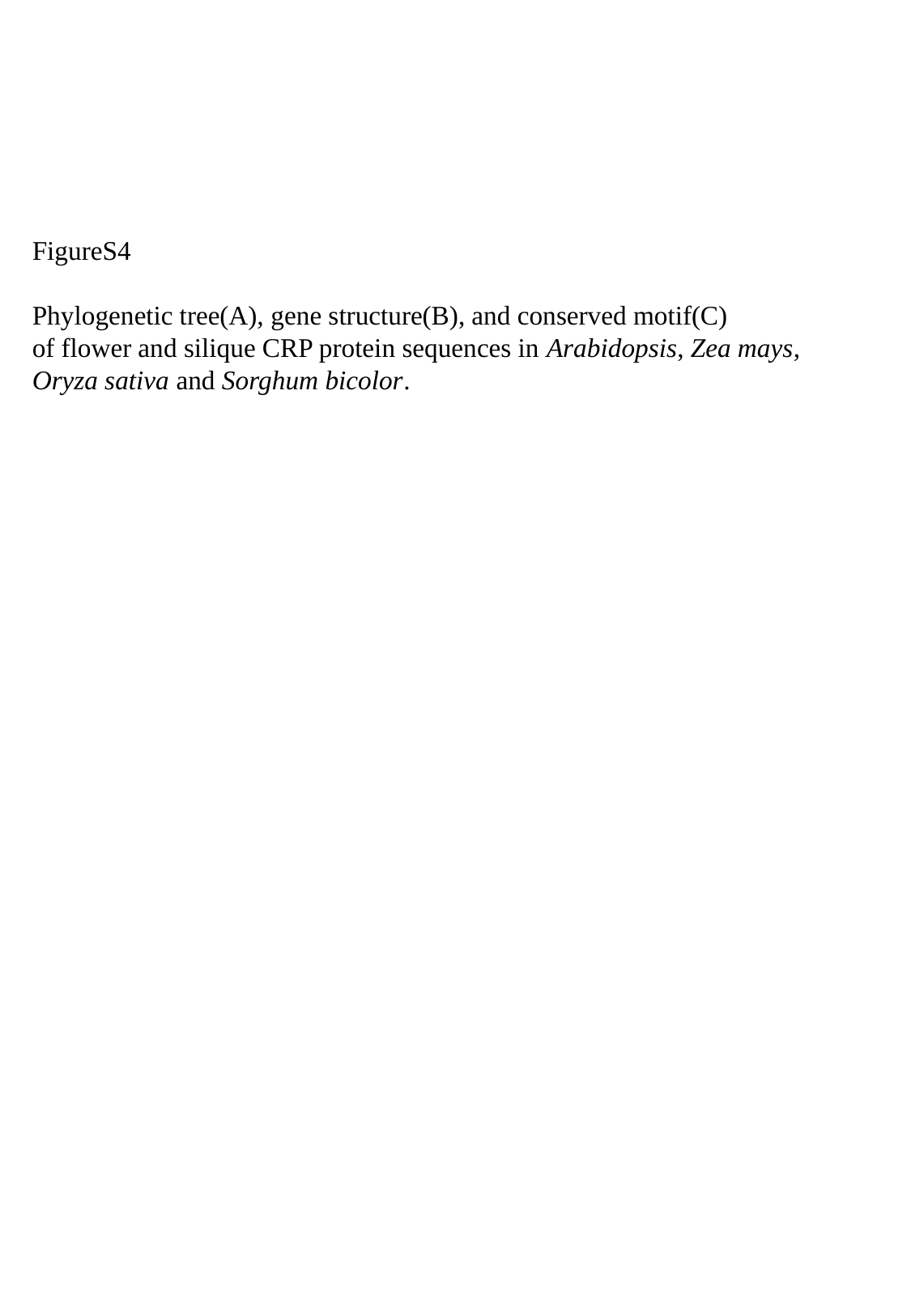

FigureS4
Phylogenetic tree(A), gene structure(B), and conserved motif(C)
of flower and silique CRP protein sequences in Arabidopsis, Zea mays,
Oryza sativa and Sorghum bicolor.

## Slide 8
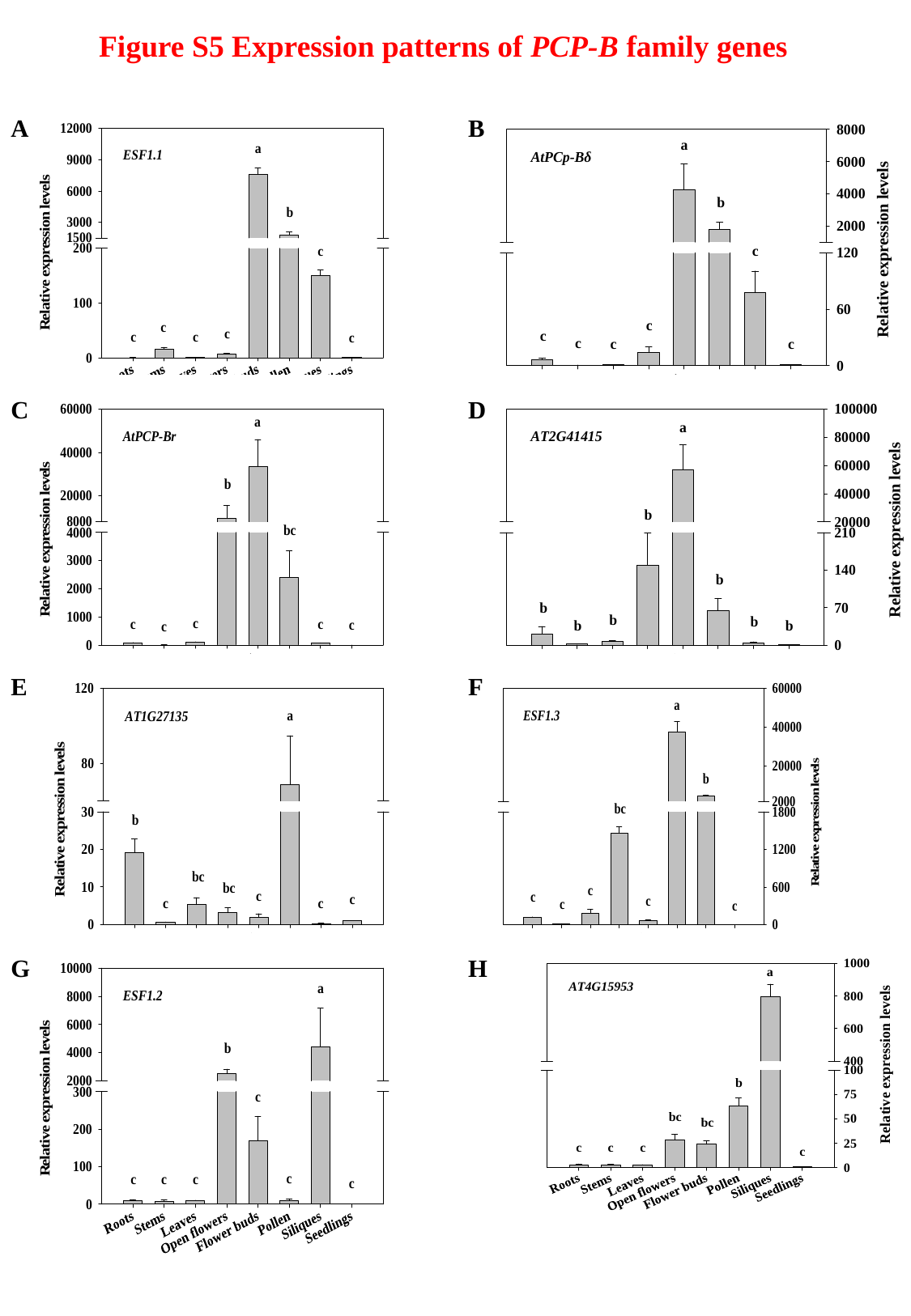

Figure S5 Expression patterns of PCP-B family genes

## Slide 9
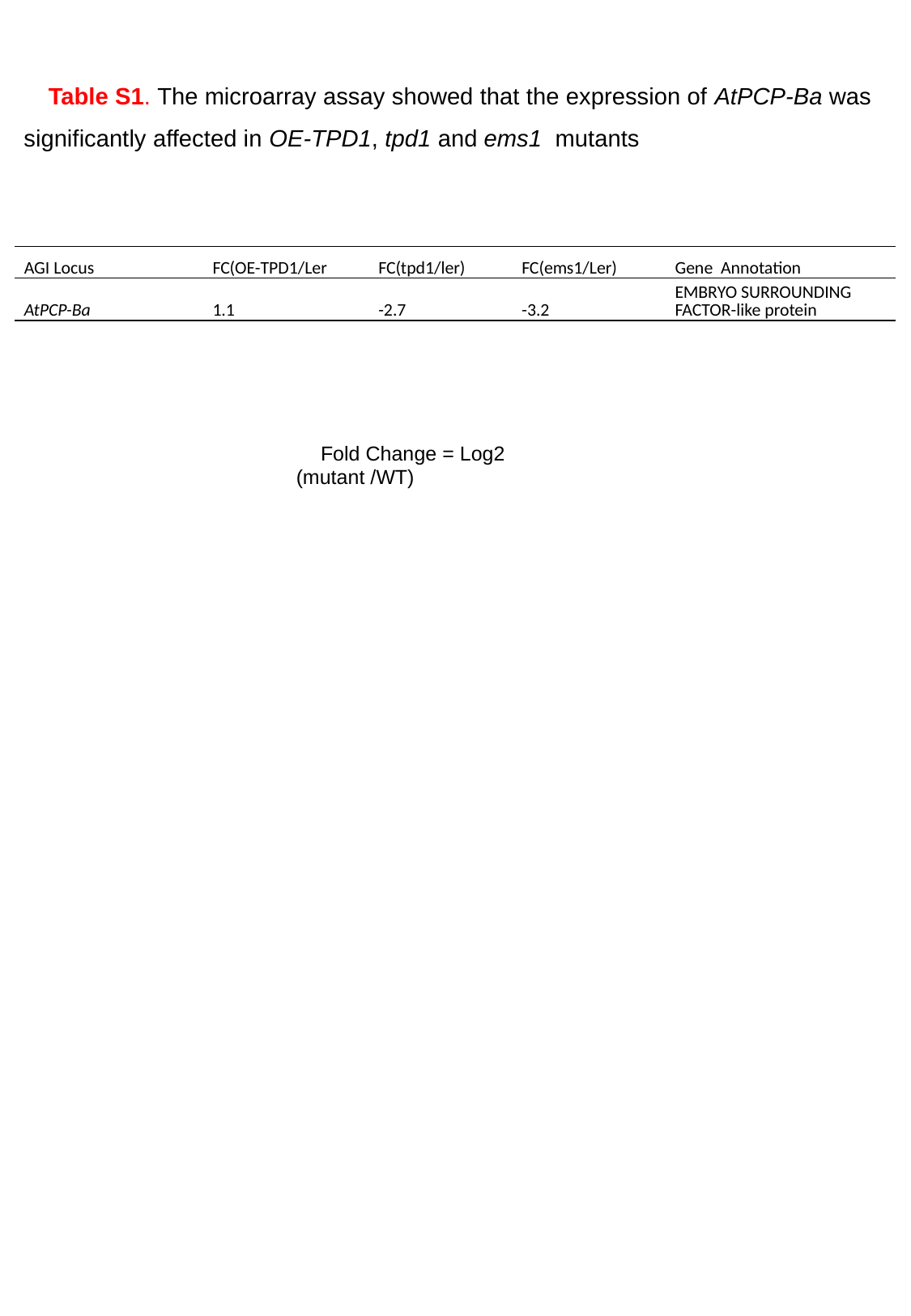

Table S1. The microarray assay showed that the expression of AtPCP-Ba was significantly affected in OE-TPD1, tpd1 and ems1 mutants
| AGI Locus | FC(OE-TPD1/Ler | FC(tpd1/ler) | FC(ems1/Ler) | Gene Annotation |
| --- | --- | --- | --- | --- |
| AtPCP-Ba | 1.1 | -2.7 | -3.2 | EMBRYO SURROUNDING FACTOR-like protein |
Fold Change = Log2 (mutant /WT)

## Slide 10
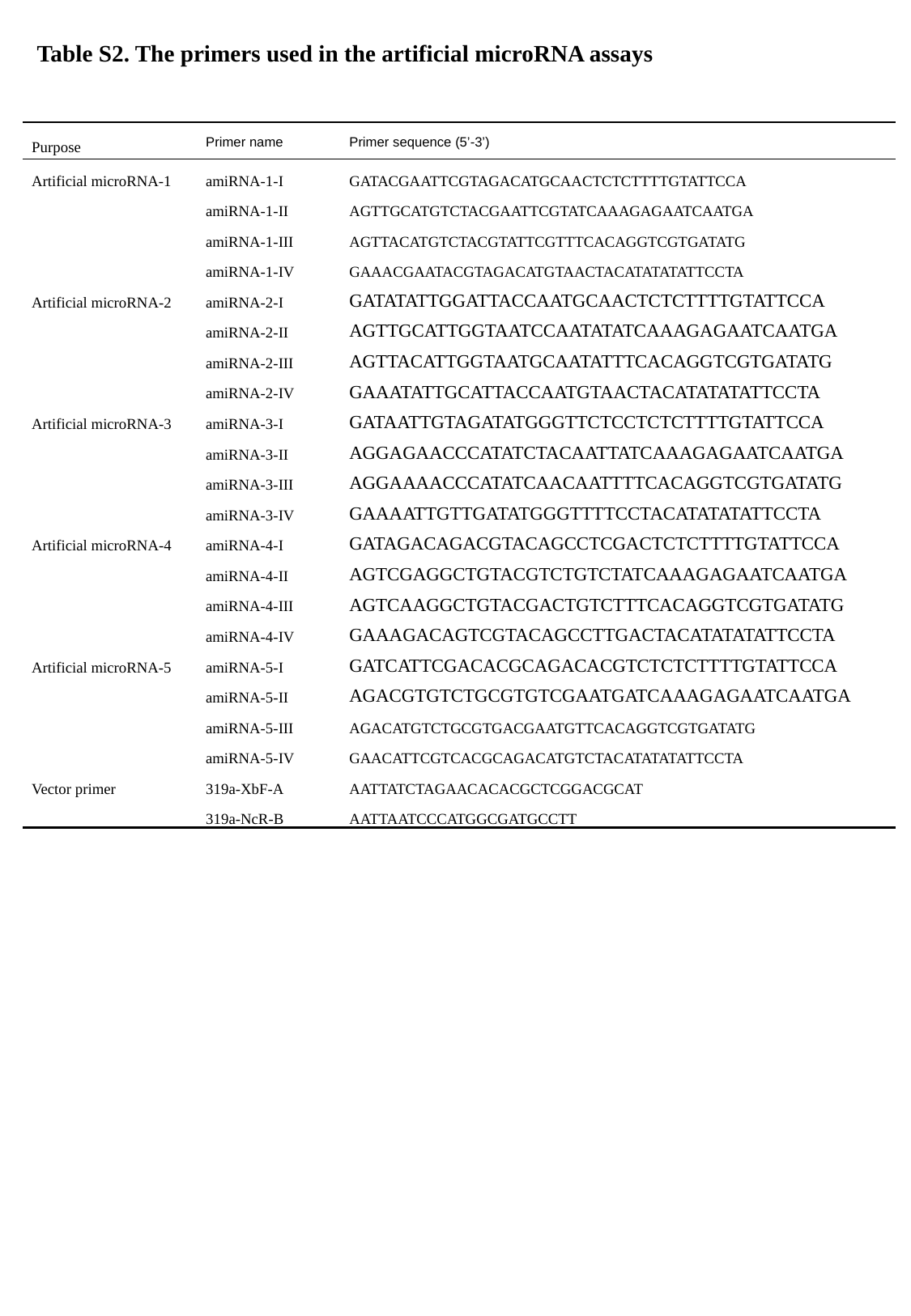

Table S2. The primers used in the artificial microRNA assays
| Purpose | Primer name | Primer sequence (5’-3’) |
| --- | --- | --- |
| Artificial microRNA-1 | amiRNA-1-I | GATACGAATTCGTAGACATGCAACTCTCTTTTGTATTCCA |
| | amiRNA-1-II | AGTTGCATGTCTACGAATTCGTATCAAAGAGAATCAATGA |
| | amiRNA-1-III | AGTTACATGTCTACGTATTCGTTTCACAGGTCGTGATATG |
| | amiRNA-1-IV | GAAACGAATACGTAGACATGTAACTACATATATATTCCTA |
| Artificial microRNA-2 | amiRNA-2-I | GATATATTGGATTACCAATGCAACTCTCTTTTGTATTCCA |
| | amiRNA-2-II | AGTTGCATTGGTAATCCAATATATCAAAGAGAATCAATGA |
| | amiRNA-2-III | AGTTACATTGGTAATGCAATATTTCACAGGTCGTGATATG |
| | amiRNA-2-IV | GAAATATTGCATTACCAATGTAACTACATATATATTCCTA |
| Artificial microRNA-3 | amiRNA-3-I | GATAATTGTAGATATGGGTTCTCCTCTCTTTTGTATTCCA |
| | amiRNA-3-II | AGGAGAACCCATATCTACAATTATCAAAGAGAATCAATGA |
| | amiRNA-3-III | AGGAAAACCCATATCAACAATTTTCACAGGTCGTGATATG |
| | amiRNA-3-IV | GAAAATTGTTGATATGGGTTTTCCTACATATATATTCCTA |
| Artificial microRNA-4 | amiRNA-4-I | GATAGACAGACGTACAGCCTCGACTCTCTTTTGTATTCCA |
| | amiRNA-4-II | AGTCGAGGCTGTACGTCTGTCTATCAAAGAGAATCAATGA |
| | amiRNA-4-III | AGTCAAGGCTGTACGACTGTCTTTCACAGGTCGTGATATG |
| | amiRNA-4-IV | GAAAGACAGTCGTACAGCCTTGACTACATATATATTCCTA |
| Artificial microRNA-5 | amiRNA-5-I | GATCATTCGACACGCAGACACGTCTCTCTTTTGTATTCCA |
| | amiRNA-5-II | AGACGTGTCTGCGTGTCGAATGATCAAAGAGAATCAATGA |
| | amiRNA-5-III | AGACATGTCTGCGTGACGAATGTTCACAGGTCGTGATATG |
| | amiRNA-5-IV | GAACATTCGTCACGCAGACATGTCTACATATATATTCCTA |
| Vector primer | 319a-XbF-A | AATTATCTAGAACACACGCTCGGACGCAT |
| | 319a-NcR-B | AATTAATCCCATGGCGATGCCTT |

## Slide 11
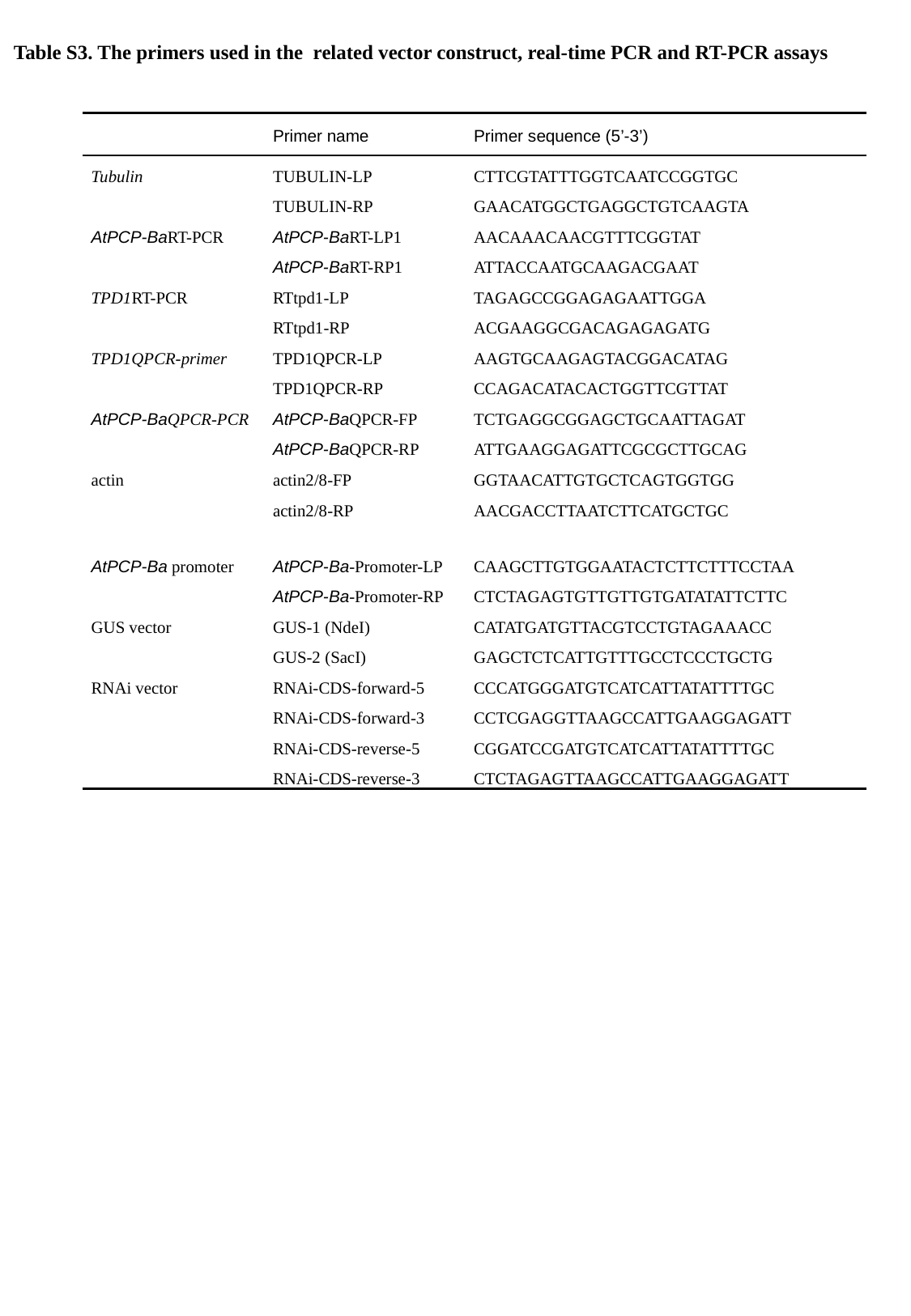

Table S3. The primers used in the related vector construct, real-time PCR and RT-PCR assays
| | Primer name | Primer sequence (5’-3’) |
| --- | --- | --- |
| Tubulin | TUBULIN-LP | CTTCGTATTTGGTCAATCCGGTGC |
| | TUBULIN-RP | GAACATGGCTGAGGCTGTCAAGTA |
| AtPCP-BaRT-PCR | AtPCP-BaRT-LP1 | AACAAACAACGTTTCGGTAT |
| | AtPCP-BaRT-RP1 | ATTACCAATGCAAGACGAAT |
| TPD1RT-PCR | RTtpd1-LP | TAGAGCCGGAGAGAATTGGA |
| | RTtpd1-RP | ACGAAGGCGACAGAGAGATG |
| TPD1QPCR-primer | TPD1QPCR-LP | AAGTGCAAGAGTACGGACATAG |
| | TPD1QPCR-RP | CCAGACATACACTGGTTCGTTAT |
| AtPCP-BaQPCR-PCR | AtPCP-BaQPCR-FP | TCTGAGGCGGAGCTGCAATTAGAT |
| | AtPCP-BaQPCR-RP | ATTGAAGGAGATTCGCGCTTGCAG |
| actin | actin2/8-FP | GGTAACATTGTGCTCAGTGGTGG |
| | actin2/8-RP | AACGACCTTAATCTTCATGCTGC |
| | | |
| AtPCP-Ba promoter | AtPCP-Ba-Promoter-LP | CAAGCTTGTGGAATACTCTTCTTTCCTAA |
| | AtPCP-Ba-Promoter-RP | CTCTAGAGTGTTGTTGTGATATATTCTTC |
| GUS vector | GUS-1 (NdeI) | CATATGATGTTACGTCCTGTAGAAACC |
| | GUS-2 (SacI) | GAGCTCTCATTGTTTGCCTCCCTGCTG |
| RNAi vector | RNAi-CDS-forward-5 | CCCATGGGATGTCATCATTATATTTTGC |
| | RNAi-CDS-forward-3 | CCTCGAGGTTAAGCCATTGAAGGAGATT |
| | RNAi-CDS-reverse-5 | CGGATCCGATGTCATCATTATATTTTGC |
| | RNAi-CDS-reverse-3 | CTCTAGAGTTAAGCCATTGAAGGAGATT |
